# Chidamide, a Histone Deacetylase Inhibitor, Combined With R‐GemOx in Relapsed/Refractory Diffuse Large B‐Cell Lymphoma (TRUST): A Multicenter, Single‐Arm, Phase 2 Trial

**DOI:** 10.1002/cam4.70919

**Published:** 2025-05-02

**Authors:** Qihua Zou, Yuchen Zhang, Hui Zhou, Yulin Lai, Yi Cao, Zhiming Li, Ning Su, Wenyu Li, Huiqiang Huang, Panpan Liu, Xu Ye, Yudan Wu, Huo Tan, Runhui Zheng, Bingyi Wu, Hui Yang, Liye Zhong, Yuhong Lu, Yang Liang, Peng Sun, Lirong Li, Yingxian Liu, Danling Dai, Yi Xia, Qingqing Cai

**Affiliations:** ^1^ State Key Laboratory of Oncology in South China, Guangdong Provincial Clinical Research Center for Cancer Sun Yat‐sen University Cancer Center Guangzhou China; ^2^ Department of Experimental Research, Sun Yat‐Sen University Cancer Center Guangzhou China; ^3^ Department of Medical Oncology Sun Yat‐sen University Cancer Center Guangzhou China; ^4^ Department of Lymphoma and Hematology Hunan Cancer Hospital Changsha China; ^5^ Department of Oncology Guangzhou Chest Hospital Guangzhou China; ^6^ Division of Lymphoma, Department of Clinical Oncology Guangdong Provincial People's Hospital Guangzhou China; ^7^ Department of Hematology The Second Affiliated Hospital of Guangzhou Medical University Guangzhou China; ^8^ Department of Hematology Sun Yat‐sen Memorial Hospital Guangzhou China; ^9^ Department of Hematology The First Affiliated Hospital of Guangzhou Medical University Guangzhou China; ^10^ Department of Hematology The Fifth Affiliated Hospital of Guangzhou Medical University Guangzhou China; ^11^ Department of Hematologic Oncology Sun Yat‐sen University Cancer Center Guangzhou China; ^12^ Department of Hematology Shunde Hospital of Southern Medical University Foshan China; ^13^ International Cancer Medical Center Jinshazhou Hospital of Guangzhou University of Chinese Medicine Guangzhou China

**Keywords:** chidamide, diffuse large B‐cell lymphoma, refractory or relapsed, R‐GemOx, transplantation‐ineligible

## Abstract

**Background:**

Histone deacetylase (HDAC) inhibitors demonstrated a synergistic anti‐tumor effect with rituximab and chemotherapy in preclinical studies on diffuse large B‐cell lymphoma (DLBCL). This phase 2 trial aimed to evaluate the efficacy and safety of chidamide, an orally active HDAC inhibitor, plus the R‐GemOx regimen for relapsed/refractory (R/R) DLBCL.

**Methods:**

Patients with transplantation‐ineligible R/R DLBCL received chidamide (20 mg, oral, days 1, 4, 8, 11, 15, and 18), rituximab (375 mg/m^2^, day 1), gemcitabine (1000 mg/m^2^, day 2), and oxaliplatin (100 mg/m^2^, day 2) in a 21‐day cycle for 6 cycles (induction phase), followed by chidamide (20 mg, oral, twice weekly on Mondays and Thursdays) until disease progression or intolerable toxicity (maintenance phase). The primary endpoint was overall response rate (ORR).

**Results:**

Between June 19, 2019 and July 5, 2022, 54 patients were enrolled. The ORR was 59.3% (95% CI: 45.0–72.4). With a median follow‐up of 38.1 months (interquartile range: 19.5–48.2), the median progression‐free survival and overall survival were 7.4 (95% CI: 5.2–14.2) and 23.9 (95% CI: 15.2‐not reached) months, respectively. The most common grade 3/4 treatment‐emergent adverse events (TEAEs) were neutropenia (40.7%), thrombocytopenia (33.3%), and leukopenia (27.8%). Whole‐exome sequencing showed that *CREBBP* mutations and *BTG2* mutations were associated with poor response and survival.

**Conclusion:**

Chidamide plus R‐GemOx demonstrated promising anti‐tumor activity with acceptable toxicities in transplantation‐ineligible R/R DLBCL patients. Patients with *CREBBP* mutations and *BTG2* mutations had inferior response and survival.

**Trial Registration:**

ClinicalTrials.gov identifier: NCT04022005

## 1 Introduction

Diffuse large B‐cell lymphoma (DLBCL) is the most common subtype of non‐Hodgkin lymphoma (NHL), accounting for approximately 30% of all NHL cases [1]. The R‐CHOP (rituximab, cyclophosphamide, vincristine, doxorubicin, and prednisone) regimen is the first‐line treatment, resulting in a cure rate of approximately 60% [1]. The pola‐R‐CHP regimen (polatuzumab vedotin plus R‐CHP) has been recommended as the standard of care in DLBCL patients with International Prognostic Index (IPI) ≥ 2 based on the result of the POLARIX trial (2‐year progression‐free survival [PFS]: 76.7% versus 70.2% in the R‐CHOP arm) [2]. However, 30%–40% of the patients are either refractory to, or relapse after treatment with the R‐CHOP regimen [3, 4].

High‐dose salvage chemotherapy followed by autologous stem‐cell transplantation (ASCT) was recommended as second‐line therapy for chemotherapy‐sensitive refractory/relapsed (R/R) DLBCL [4, 5, 6]. The overall cure rate is 25%–35%, but 50% of the patients are not candidates for ASCT due to age, underlying diseases, or inadequate response to salvage therapy [4, 7]. Based on the superior event‐free survival (EFS) with chimeric antigen receptor‐T (CAR‐T) therapy versus high‐dose salvage chemotherapy plus ASCT in patients with primary refractory DLBCL or those who relapsed within 12 months of first‐line treatment in the TRANSFORM and ZUMA‐7 trials [8, 9]. CAR‐T therapy has also been recommended as a standard second‐line treatment. However, its application is limited by economic considerations, potential adverse events, and manufacturing limitations [10]. In the SCHOLAR‐1 study, the overall response rate (ORR) to subsequent rituximab‐based chemotherapy in refractory DLBCL patients was 26%, with a median overall survival (OS) of 6.3 months [11].

Low level of histone acetylation has been implicated in hematological malignancies, including lymphoma [12, 13], leukemia [14], and multiple myeloma [15]. In DLBCL, histone acetylation level was lower in activated B‐cell‐like (ABC) subtype with poorer prognosis than in GCB subtype [16]. Inactivating mutations in histone acetyltransferase (HAT) genes *CREBBP* and *EP300* were identified in approximately 30% of DLBCL cases [12, 13], whereas mutations in other HAT family members (*TIP60*, *MOZ*, *CLOCK*, *NCOA2*, *NCOA3*) exhibited lower frequencies (< 5%) [17, 18]. *CREBBP/EP300* mutations promoted the tumor progression of DLBCL through constitutive activation of the BCL6 oncoprotein, functional inactivation of p53 tumor suppressor activity, and activation of the NOTCH pathway by suppressing histone acetylation [13, 19].

Upregulation of histone deacetylase (HDAC) 1, 2, 3, and 6 was observed in DLBCL tumor tissues, suggesting their potential as therapeutic targets for DLBCL [20, 21, 22, 23]. HDAC inhibitor monotherapy has shown promising efficacy in R/R DLBCL with the ORR ranging from 19% to 29% in phase 2 trials [24, 25]. Chidamide, an orally active benzamide class of HDAC inhibitor, is more selective to HDAC 1, 2, 3, and 10 than vorinostat and panobinostat [26, 27]. In cultured DLBCL cells, chidamide and gemcitabine/oxaliplatin produced synergistic effects in inducing cell cycle arrest and promoting cell apoptosis [28]. HDAC inhibitor could sensitize rituximab‐resistant DLBCL cells by upregulating CD20 expression through promoting CD20 promoter acetylation and Sp1 recruitment [29]. In a preclinical study, chidamide induced apoptosis of DLBCL cells by suppressing the HDACs/STAT3/Bcl‐2 pathway [30]. Chidamide has higher anti‐tumor activity in *TP53*‐mutant DLBCL cells compared to *TP53*‐wildtype DLBCL cells and reduced the mRNA and protein levels of mutant *TP53* [31]. The potential preferential activities of HDAC inhibitors need further investigation due to the high heterogeneity of DLBCL.

Based on these findings, we conducted a multicenter, single‐arm, phase 2 trial (TRUST trial) to evaluate the efficacy and safety of chidamide in combination with the R‐GemOx (CR‐GemOx) regimen for transplantation‐ineligible R/R DLBCL patients.

## 2 Materials and Methods

### 2.1 Study Design and Participants

The trial is a multicenter, single‐arm, phase 2 trial conducted in 8 centers in China. Adult DLBCL patients (18–75 years of age) who were refractory to or relapsed after anthracycline‐based systemic regimens and were not candidates for ASCT were eligible. Additional inclusion criteria included Eastern Cooperative Oncology Group performance status (ECOG PS) of 0–1, adequate organ function, and at least one measurable lesion. Key exclusion criteria included double‐hit or triple‐hit DLBCL, central nervous system (CNS) involvement, and treatment with gemcitabine within the past 6 months or HDAC inhibitor at any time. The full inclusion and exclusion criteria are shown in Appendix S1.

The trial was approved by the Ethics Committee of Sun Yat‐sen University Cancer Center and conducted in accordance with the Declaration of Helsinki and the International Conference on Harmonization Good Clinical Practice guidelines. Written informed consent was obtained from all patients.

### 2.2 Procedures

Eligible patients received the CR‐GemOx regimen for 6 cycles (induction phase). Each cycle lasted for 3 weeks and included: (1) 20 mg chidamide p.o. on days 1, 4, 8, 11, 15, and 18; (2) 375 mg/m^2^ rituximab via intravenous infusion on day 1; (3) 1000 mg/m^2^ gemcitabine and 100 mg/m^2^ oxaliplatin via intravenous infusion on day 2. Tumor response was evaluated every 6 weeks during the induction phase by computed tomography (CT) or positron emission tomography‐computed tomography (PET‐CT) in accordance with the 2014 Lugano criteria [32]. Patients who achieved complete or partial response proceeded to maintenance therapy with chidamide (20 mg, oral, twice weekly on Mondays and Thursdays) until disease progression or intolerable toxicity. CT was performed every 8 weeks in the maintenance phase. In patients with severe nausea and vomiting, 5‐HT_3_ receptor blockers were given prior to chidamide.

Treatment‐emergent adverse events (TEAEs) were recorded at every visit and graded using the National Cancer Institute Common Terminology Criteria for Adverse Events version 5.0 (NCI‐CTCAE 5.0). Serious adverse events (SAEs) were defined as AEs that led to or prolonged hospitalization, life‐threatening event, death, or permanent disability.

### 2.3 Endpoints

The primary endpoint was ORR, defined as the proportion of patients who achieved CR or partial response (PR). Secondary endpoints included disease control rate (DCR), defined as the proportion of patients who achieved CR, PR, or SD; time to response (TTR), calculated from the time of enrollment until the first response; duration of response (DOR), defined as the time from the first CR or PR to the first documented progressive disease (PD) or death, whichever occurred earlier; progression‐free survival (PFS), defined as the time from the date of enrollment until either PD or death; OS, defined as the time from the date of enrollment until death; and TEAEs. Exploratory endpoint was the relationship between gene mutations and efficacy measures (ORR, PFS, and OS).

### 2.4 Methods for Exploratory Analyses

Cell of origin (COO) subtype of DLBCL was classified according to the immunohistochemistry‐based Hans algorithm [33]. Levels of c‐myc and bcl‐2 were determined with immunohistochemistry. Positive thresholds of c‐myc and bcl‐2 were defined as cut‐off values of 40% and 50%, respectively. IPI was assessed at the time of enrollment. Whole‐exome sequencing (WES) and RNA sequencing (RNAseq) were performed on 20 and 17 pre‐treatment formalin‐fixed paraffin‐embedded (FFPE) tumor tissues, respectively. The detailed information of WES and RNAseq is presented in Supplementary methods in Supporting Information S2.

### 2.5 Statistical Analysis

Sample size requirement was estimated using PASS version 15 software based on the following assumptions: (1) ORR with R‐GemOx regimen at 44% [34]; (2) ORR with CR‐GemOx at 64% (20% absolute increase relative to R‐GemOx); (3) 80% power and two‐sided alpha of 0.05. The calculation yielded 48 subjects. Assuming 10% drop‐out, we planned to enroll 54 patients.

The intention‐to‐treat (ITT) population consisted of all enrolled patients. The efficacy‐evaluable population included all patients who received the study treatment and had at least one postbaseline response assessment. The baseline clinical characteristics, ORR, DCR, and safety were summarized by descriptive statistics. The two‐sided 95% confidence interval (CI) for response was calculated by the Clopper‐Pearson exact method. Survival outcomes of DOR, PFS, OS, and TTR were estimated by the Kaplan–Meier method, and compared using a log‐rank test. Categorical variables were analyzed using the chi‐square test or Fisher's exact test, as appropriate. *p* < 0.05 (2‐sided) was considered statistically significant. Statistical analyses were performed R version 4.3.0 software for windows.

## 3 Results

### 3.1 Patient Characteristics

**FIGURE 1:**
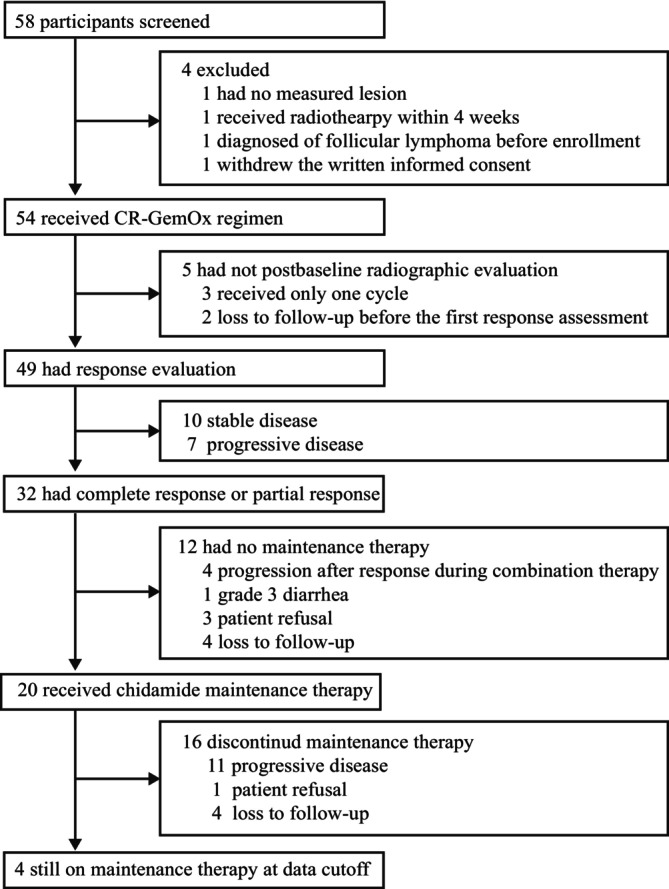
Patient flow through the trial.

A total of 58 patients were screened from August 12, 2019, to July 5, 2022; 54 patients were enrolled and received at least one treatment cycle (Figure 1). The median age was 65 (interquartile range [IQR]: 55–67) years. The median number of previous lines of therapy was 1 (range: 1–4). Baseline characteristics are listed in Table 1. Five patients were not evaluable for response (3 received only one treatment cycle, 2 were lost to follow‐up before the first postbaseline response assessment).

**TABLE 1:** Baseline demographics and disease characteristics (*n* = 54).

	Total (*N* = 54)
Age, years	
Median (range)	65 (25–74)
Sex	
Male	29 (53.7%)
Female	25 (46.3%)
ECOG performance status	
0	29 (53.7%)
1	25 (46.3%)
Disease status^a^	
Relapsed	22 (40.7%)
Refractory	32 (59.3%)
Response to first‐line therapy	
Complete response	29 (53.7%)
Partial response	21 (38.9%)
Stable disease	2 (3.7%)
Progressive disease	2 (3.7%)
Cell of origin by immunohistochemistry	
GCB	16 (29.6%)
Non‐GCB	38 (70.4%)
Ann Arbor stage	
I or II	15 (27.8%)
III or IV	39 (72.2%)
Lactate dehydrogenase	
Normal	27 (50.0%)
Elevated	27 (50.0%)
Extranodal sites of involvement	
0–1	30 (55.6%)
≥ 2	24 (44.4%)
Bone marrow of involvement	
No	46 (85.2%)
Yes	8 (14.8%)
International Prognostic Index	
0 or 1	17 (31.5%)
2	14 (25.9%)
3	17 (31.5%)
4 or 5	6 (11.1%)
c‐myc/bcl‐2 double‐expression	
No	38 (70.4%)
Yes	16 (29.6%)
CD‐5	
Negative	42 (77.8%)
Positive	9 (16.7%)
Unknown	3 (5.5%)
Previous lines of systemic therapy	
Median (range)	1 (1–4)
1	36 (66.7%)
2	15 (27.8%)
3	2 (3.7%)
4	1 (1.8%)
Prior rituximab	
Yes	50 (92.6%)
No	4 (7.4%)

*Note:* Data are shown as number (%) unless otherwise specified.

Abbreviations: DLBCL, diffuse large B‐cell lymphoma; ECOG, Eastern Cooperative Oncology Group; GCB, germinal center B cell.

^a^ Refractory DLBCL was defined as incomplete response to (*n* = 25), or recurrence with 12 months of first‐line therapy (*n* = 7). Relapsed DLBCL was defined as recurrence after 12 months of first‐line therapy (*n* = 22).

### 3.2 Treatment

**FIGURE 2:**
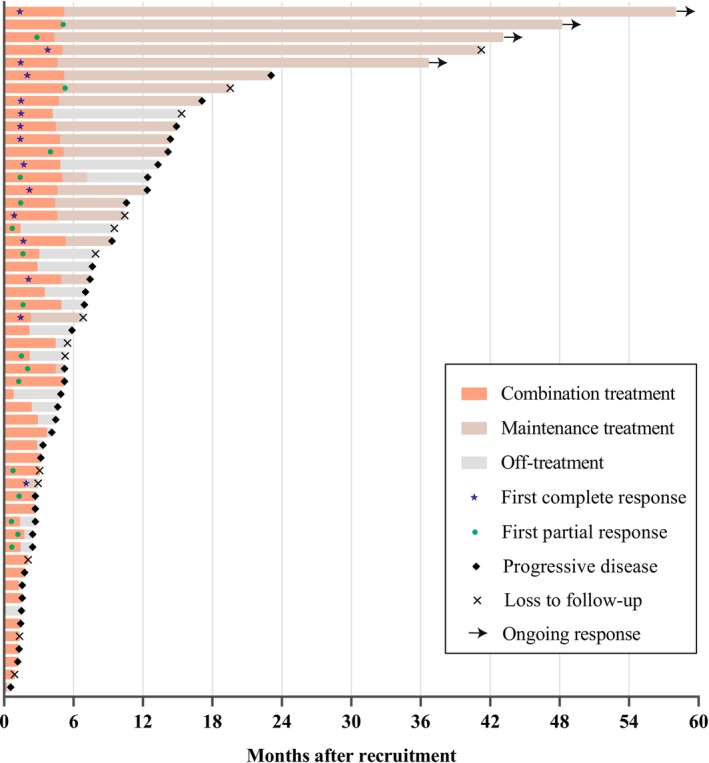
Swimmer plot of patients.

The median number of CR‐GemOx cycles was 4 (IQR: 2–6 cycles). Among the 32 patients who achieved CR or PR, 20 proceeded to maintenance therapy with chidamide (Figure 1). At the time of data cutoff (September 3, 2024), 4 of 20 patients were still receiving chidamide maintenance therapy (Figure 1) and had ongoing responses (2 and 2 for CR and PR, respectively) (Figure 2).

### 3.3 Efficacy

**FIGURE 3:**
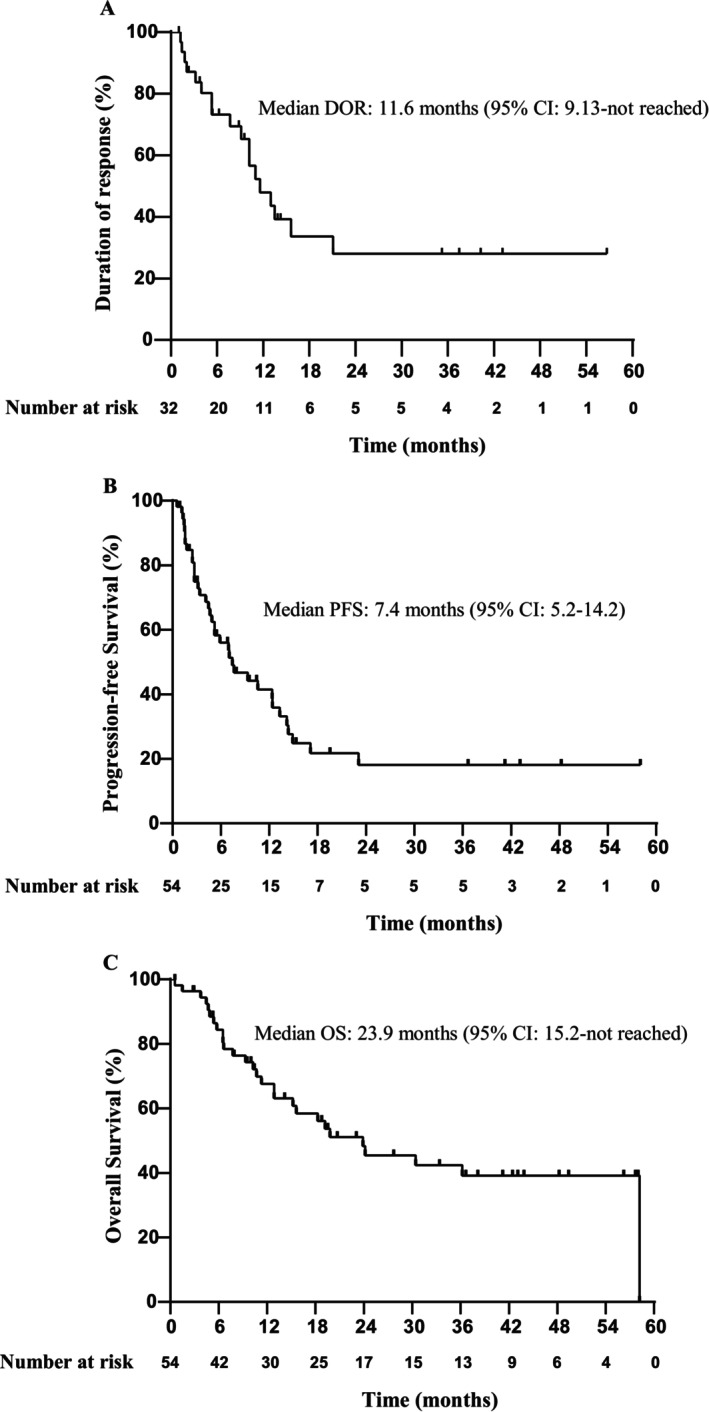
Kaplan–Meier survival curves. (A) Duration of response in the 31 responding patients. (B) Progression‐free survival of 54 patients. (C) Overall survival of 54 patients.

Number of the patients who achieved CR, PR, and SD was 15, 17, and 10, respectively. In the ITT population (*n* = 54), the ORR was 59.3% (32/54; 95% CI: 45.0–72.4), and DCR was 77.8% (42/54; 95% CI: 64.4–88.0). The rate of CR and PR was 27.8% (15/54; 95% CI: 16.5–41.6) and 31.5% (17/54; 95% CI: 19.5–45.6), respectively (Table 2). In the efficacy‐evaluable population (*n* = 49), the ORR was 65.3% (32/49; 95% CI: 50.4–78.3) (Table 2). Subgroup analyses showed similar ORR across subgroups stratified by disease stage, number of previous lines of therapy, COO subtype, CD5 status, double expression, or disease category (Figure S1). The median TTR was 1.47 months (95% CI: 1.4–1.9). The median DOR in responding patients was 11.6 months (95% CI: 9.13–not reached) (Figure 3A).

**TABLE 2:** Anti‐tumor activity.

Response evaluation	Intention‐to‐treat population (*n* = 54)	Efficacy‐evaluable population (*n* = 49)
Objective response rate, *n* (%)	32 (59.3)	32 (65.3)
95% CI	45.0–72.4	50.4–78.3
Disease control rate, *n* (%)	42 (77.8)	42 (85.7)
95% CI	64.4–88.0	72.8–94.1
Best overall response, *n* (%, 95% CI)		
Complete response	15 (27.8, 16.5–41.6)	15 (30.6, 18.3–45.4)
Partial response	17 (31.5, 19.5–45.6)	17 (34.7, 21.7–49.6)
Stable disease	10 (18.5, 9.3–31.4)	10 (20.4, 10.2–34.3)
Progressive disease	7 (13.0, 5.4–24.9)	7 (14.3, 5.9–27.2)
Not evaluable	5 (9.2, 3.1–20.3)	—

*Note:* Data are shown as number (%) or number (%, 95% CI). Responses were assessed in accordance with the revised 2014 Lugano criteria for response assessment of lymphoma.

### 3.4 Survival

With a median follow‐up of 38.1 months (IQR: 19.5–48.2), 36 (66.7%) patients had a PFS event, and 28 (51.9%) patients had died. The median PFS and OS were 7.4 months (95% CI: 5.2–14.2) and 23.9 months (95% CI: 15.2–not reached), respectively (Figure 3B,C). The 12‐month and 24‐month PFS rates were 41.5% (95% CI: 29.3–58.7) and 15.5% (95% CI: 7.3–33.3), respectively. The 12‐month and 24‐month OS rates were 67.6% (95% CI: 55.7–82.1) and 48.3% (95% CI: 35.5–65.6), respectively.

Among the 36 patients with PFS events, 18 (50.0%) received subsequent treatments, including CAR‐T cell therapy, bispecific antibody, antibody‐drug conjugate, BTK inhibitor, and PI3Kδ inhibitor (Table S1).

In the post hoc exploratory univariate analyses, elevated lactate dehydrogenase (LDH) (median PFS: 4.6 versus 13.3 months; HR [hazard ratio]: 2.5; 95% CI: 1.3–4.9; *p* = 0.007), involvement of ≥ 2 extranodal sites (median PFS: 4.6 versus 13.3 months; HR: 2.2; 95% CI: 1.1–4.3; *p* = 0.018), IPI ≥ 2 (median PFS: 5.2 versus 14.9 months; HR: 2.2; 95% CI: 1.0–4.8; *p* = 0.036), and previous lines of therapy ≥ 2 (median PFS: 5.2 versus 9.3 months; HR: 2.1; 95% CI: 1.1–4.2; *p* = 0.023) were associated with shorter PFS in univariate analyses (Figure S2A–D). No significant differences between PFS and age, gender, ECOG PS, stage, COO subtype, disease status, CD5 status, double expression, c‐myc level were observed (Figure S3A–I).

In the univariate analyses, CD5 positive (median OS: 10.7 versus 36.2 months; HR: 2.4; 95% CI: 1.0–5.8; *p* = 0.046), and involvement of ≥ 2 extranodal sites (median OS: 15.6 months versus not reached; HR: 2.2; 95% CI: 1.0–4.8; *p* = 0.036) were associated with shorter OS (Figure S4A,B). There were no significant differences between OS and age, gender, ECOG PS, stage, COO subtype, disease status, LDH, double expression, previous lines of therapy, IPI, and c‐myc level (Figure S5A–K).

### 3.5 Treatment‐Emergent Adverse Events

Any grade TEAEs were observed in 96.3% (52/54) of the patients, and the majority of TEAEs were grade 1–2. The TEAEs with ≥ 20% frequency included leukopenia (*n* = 35, 64.8%), neutropenia (*n* = 34, 63.0%), thrombocytopenia (*n* = 31, 57.4%), anemia (*n* = 31, 57.4%), fatigue (*n* = 21, 38.9%), and nausea (*n* = 12, 22.2%) (Table 3). The most common grade 3 or more TEAEs included neutropenia (*n* = 22, 40.7%), thrombocytopenia (*n* = 18, 33.3%), and leukopenia (*n* = 15, 27.8%) (Table 3). All TEAEs were reversible upon dose delay or reduction. Three patients had febrile neutropenia but recovered within 1 week after treatment with granulocyte colony‐stimulating factor and intravenous antibiotics treatment. No bleeding events were reported. Grade ≥ 3 TEAEs that occurred in ≥ 5% of patients included neutropenia (*n* = 4, 20.0%), thrombocytopenia (*n* = 3, 15.0%), and diarrhea (*n* = 1, 5.0%) during the chidamide maintenance period.

**TABLE 3:** Treatment‐emergent adverse events (*n* = 54).

	Any grade	Grade 1–2	Grade 3	Grade 4
Hematological events				
Leukopenia	35 (64.8%)	20 (37.0%)	9 (16.7%)	6 (11.1%)
Neutropenia	34 (63.0%)	12 (22.2%)	14 (25.9%)	8 (14.8%)
Thrombocytopenia	31 (57.4%)	13 (24.1%)	6 (11.1%)	12 (22.2%)
Anemia	31 (57.4%)	27 (50.0%)	4 (7.4%)	0
Febrile neutropenia	3 (5.6%)	0	0	3 (5.6%)
Non‐hematological events				
Fatigue	21 (38.9%)	21 (38.9%)	0	0
Nausea	12 (22.2%)	12 (22.2%)	0	0
Liver dysfunction	9 (16.7%)	9 (16.7%)	0	0
Electrolyte disorders	8 (14.8%)	8 (14.8%)	0	0
Diarrhea	7 (13.0)	5 (9.3%)	2 (3.7%)	0
Constipation	5 (9.3%)	5 (9.3%)	0	0
Rash	5 (9.3%)	5 (9.3%)	0	0
Elevated triglyceride	4 (7.4%)	3 (5.6%)	1 (1.9%)	0
Arrhythmias	3 (5.6%)	3 (5.6%)	0	0
Renal dysfunction	3 (5.6%)	3 (5.6%)	0	0
Peripheral neuropathy	3 (5.6%)	3 (5.6%)	0	0
Pneumonia	1 (1.9%)	0	1 (1.9%)	0
Heart failure	1 (1.9%)	0	1 (1.9%)	0
Herpes zoster	1 (1.9%)	1 (1.9%)	0	0

*Note:* Data are shown as number (%).

One patient (1.9%) discontinued CR‐GemOx treatment due to pneumonia and heart failure, and 1 patient (1.9%) discontinued chidamide maintenance therapy due to diarrhea. Dose reduction or interruption of chidamide occurred in 19 patients (35.2%), due to neutropenia (*n* = 10, 18.5%), thrombocytopenia (*n* = 7, 13.0%), diarrhea (*n* = 1, 1.9%), and digestive tract reaction (*n* = 1, 1.9%). SAE (pneumonia and heart failure) was reported in 1 patient (1.9%). There were no treatment‐related deaths.

### 3.6 Genomic Mutation Profile and Transcriptomic Analyses

**FIGURE 4:**
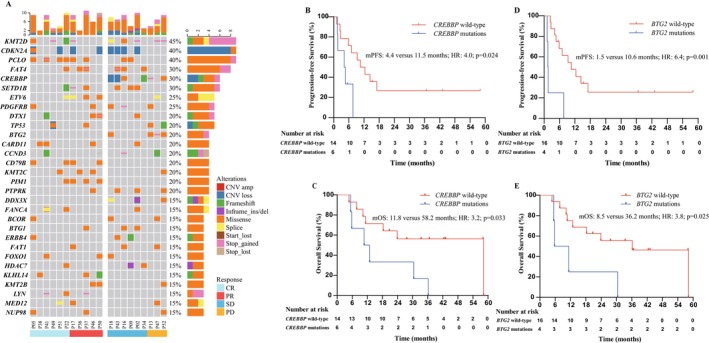
Genomic mutation profile. (A) Heatmap of genes. (B) PFS in the patients with versus without *CREBBP* mutations. (C) OS in the patients with versus without *CREBBP* mutations. (D) PFS in the patients with versus without *BTG2* mutations. (E) OS in the patients with versus without *BTG2* mutations. CR, complete response; HR, hazard ratio; PD, progressive disease; PFS, progression‐free survival; PR, partial response; SD, stable disease.

WES was conducted in 20 patients with available FFPE tissues at baseline. Patients were classified using the LymphGem classifier to: 1 A53 subtype, 1 ST2 subtype, 1 EZB subtype, 1 BN2/MCD subtype, 2 BN2 subtypes, 3 MCD subtypes, and 11 other subtypes [35]. The most frequently mutated genes included *KMT2D* (45.0%), *CDKN2A* (40.0%), *PCLO* (40.0%), *FAT4* (30.0%), *CREBBP* (30.0%), and *SETD1B* (30.0%) (Figure 4A). Treatment response and survival outcome differed significantly between patients with versus without *CREBBP* mutations (ORR: 0% versus 78.6%, *p* = 0.002; median PFS: 4.4 versus 11.5 months, HR: 4.0, 95% CI: 1.1–14.7, *p* = 0.024; median OS: 11.8 versus 58.2 months, HR: 3.2, 95% CI: 1.0–10.1, *p* = 0.033) (Figure 4B,C), as well as between patients with versus without *BTG2* mutations (ORR: 0% versus 68.8%, *p* = 0.026; median PFS: 1.5 versus 10.6 months, HR: 6.4, 95% CI: 1.7–23.3, *p* = 0.001; median OS: 8.5 versus 36.2 months, HR: 3.8, 95% CI: 1.1–13.1, *p* = 0.025) (Figure 4D,E).

RNAseq analysis was conducted in 10 responding and 7 non‐responding patients. Upregulated genes in responding vs. non‐responding patients included *HLA‐DRB5*, *CKS2*, *IPO7*, *MARS2*, *RP11‐793H13.8*, *VDAC1*, *TGDS*, *RP11‐155B2.1*, *GARS*, and *PABPC4* (Figure S6A,B). Down‐regulated genes included *RNU5D‐1*, *MBP*, *MYO7A*, *PNPLA4*, *CTB‐176F20.3*, *LEF1*, *ANKRD20A17P*, *ZCCHC2*, *SNORA37*, and *RAB15* (Figure S6A,B).

## 4 Discussion

The TRUST trial is a prospective study evaluating the anti‐tumor activity, safety profiles, and potential biomarkers of combining chidamide and R‐GemOx regimen in R/R DLBCL patients who were not candidates for ASCT. Our results revealed the promising efficacy and manageable toxicities of the CR‐GemOx regimen. The biomarker analysis indicated an association between *CREBBP* and *BTG* mutations with poor ORR and PFS.

The ORR and OS of CR‐GemOx in this trial were numerically higher than R‐Gemox reported by López et al. [34], despite a higher proportion of refractory patients (59.3% versus 40.6%) and prior exposure to rituximab (92.6% versus 25.0%) in this trial. The ORR in this trial was numerically similar to that reported for R‐GemOx by Mounier et al. [36], despite a higher percentage of patients with prior exposure to rituximab (92.6% versus 63.3%). The ORR in primary refractory DLBCL patients who met SCHOLAR‐1 criteria in this trial was 50.0% (2/4; CR in 1 patients). The patients of the SCHOLAR‐1 study were more pretreated and received rituximab‐based regimens as previous therapy, and double and triple hit B‐cell lymphoma were not excluded, which may contribute to shorter PFS [11].

The ORR reported in this trial (59.3%) was comparable to that reported for most regimens used in transplantation‐ineligible R/R DLBCL patients, including mosunetuzumab plus polatuzumab vedotin (59.2%) [37], tafasitamab plus lenalidomide (60.0%) [38], and magrolimab (an anti‐CD47 antibody) plus R‐GemOx (51.5%) [39], but higher than that reported for the R‐Gemox group (40.7%) [40]. In comparison to the Glofit‐GemOx regimen, CR‐GemOx in this trial was inferior in both ORR (59.3% vs. 68.3%) and PFS (7.4 vs. 14.4 months) [40]. However, the use of glofitamab may be limited by economic factors and the adverse events such as serious infections, cytokine release syndrome, and neurological adverse events. Nevertheless, caution must be exercised when comparing results from different trials.

The CR‐GemOx regimen was well tolerated in this trial. All TEAEs were reversible upon dose delay or reduction, adding support to the safety profile of CR‐GemOx. The most common grade ≥ 3 TEAE was neutropenia (40.7%), similar to that reported for R‐GemOx by López et al. (43%) [34] and El et al. (43%) [41], and lower than that reported for R‐GemOx by Mounier et al. (73%) [36]. Of note, the rate of SAE in this trial (1.9%) was lower than that reported for R‐GemOx by Mounier et al. (40%) [36]. Consistent with the previous studies [42, 43], the most common hematologic toxicities during chidamide monotherapy maintenance were grade ≥ 3 neutropenia (20.0%) and thrombocytopenia (15.0%). These comparisons should be interpreted with caution due to different timing of study and differences in the use of primary prophylaxis with colony‐stimulating factors.

The frequency of *CREBBP* mutation in this trial (30%) was consistent with previous studies in R/R DCBCL patients [44, 45]. Mutation of *CREBBP* plays a dual role in DLBCL. In a preclinical study, inactivating *CREBBP* increased the sensitivity of DLBCL cells to chidamide [46]. However, another study revealed that *CREBBP* mutations promote the proliferation of B‐lymphoma cells and the polarization of tumor‐related macrophages to the M2 phenotype by inhibiting histone acetylation [19]. It has also been reported that *CREBBP* mutation was associated with poor PFS [47]. Consistent with a phase 2 trial showing that only 1 of 12 patients carrying *CREBBP* inactivating mutations responded to panobinostat (a HDAC inhibitor) in R/R DLBCL [24], none of the 6 patients with *CREBBP* inactivation mutations in this trial responded to the CR‐GemOx regimen. Poorer PFS in patients with versus without *BTG2* mutations in this trial was also consistent with previous results [48]. These findings suggest that *CREBBP* and *BTG* mutations could serve as potential predictive molecular biomarkers for inferior response to HDAC inhibitor combined with chemotherapy.

In a single‐arm phase 2 trial in 49 elderly DLBCL patients, chidamide plus R‐CHOP demonstrated promising efficacy with a CR of 86% and manageable toxicity as first‐line therapy [49]. In a phase III randomized trial in DLBCL patients with c‐myc/bcl2 double expression, chidamide plus R‐CHOP resulted in a higher CR rate (73.0% versus 61.8%) and 24‐month EFS rate (58.9% versus 46.2%) compared with the control (R‐CHOP plus placebo) [50]. These results further support the combination of chidamide with rituximab‐based regimen in DLBCL.

This trial has several limitations. First, the trial did not include a comparator arm. Randomized controlled trials are needed to validate the efficacy of the CR‐GemOx regimen. Second, the sample size for genomic and transcriptomic analysis was relatively small, so further investigations are warranted to identify and validate specific genetic alterations that can predict response to the HDAC inhibitor plus chemotherapy.

In conclusion, this trial demonstrated promising anti‐tumor activity and a manageable safety profile of chidamide in combination with R‐GEMOX in transplantation‐ineligible R/R DLBCL patients. Patients with *CREBBP* mutations and *BTG2* mutations had inferior response and survival.

## Author Contributions

**Qihua Zou:** conceptualization (equal), data curation (equal), formal analysis (equal), methodology (equal), project administration (equal), software (equal), validation (equal), visualization (equal), writing – original draft (equal), writing – review and editing (equal). **Yuchen Zhang:** data curation (equal), formal analysis (equal), methodology (equal), project administration (equal), validation (equal), visualization (equal), writing – original draft (equal), writing – review and editing (equal). **Hui Zhou:** data curation (equal), investigation (equal), resources (equal), writing – review and editing (equal). **Yulin Lai:** data curation (equal), writing – original draft (equal), writing – review and editing (equal). **Yi Cao:** data curation (supporting), writing – review and editing (supporting). **Zhiming Li:** investigation (supporting), resources (supporting), writing – review and editing (supporting). **Ning Su:** conceptualization (supporting), investigation (supporting), project administration (supporting), resources (supporting), writing – review and editing (supporting). **Wenyu Li:** investigation (supporting), resources (supporting), writing – review and editing (supporting). **Huiqiang Huang:** investigation (supporting), resources (supporting), writing – review and editing (supporting). **Panpan Liu:** investigation (supporting), resources (supporting), writing – review and editing (supporting). **Xu Ye:** investigation (supporting), resources (supporting), writing – review and editing (supporting). **Yudan Wu:** investigation (supporting), resources (supporting), writing – review and editing (supporting). **Huo Tan:** investigation (supporting), resources (supporting), writing – review and editing (supporting). **Runhui Zheng:** investigation (supporting), resources (supporting), writing – review and editing (supporting). **Bingyi Wu:** investigation (supporting), resources (supporting), writing – review and editing (supporting). **Hui Yang:** investigation (supporting), resources (supporting), writing – review and editing (supporting). **Liye Zhong:** investigation (supporting), resources (supporting), writing – review and editing (supporting). **Yuhong Lu:** investigation (supporting), resources (supporting), writing – review and editing (supporting). **Yang Liang:** investigation (supporting), resources (supporting), writing – review and editing (supporting). **Peng Sun:** investigation (supporting), resources (supporting), writing – review and editing (supporting). **Lirong Li:** data curation (supporting), project administration (supporting), writing – review and editing (supporting). **Yingxian Liu:** data curation (supporting), project administration (supporting), writing – review and editing (supporting). **Danling Dai:** writing – original draft (equal), writing – review and editing (equal). **Yi Xia:** conceptualization (equal), investigation (equal), methodology (equal), project administration (equal), resources (equal), supervision (equal), writing – review and editing (equal). **Qingqing Cai:** conceptualization (lead), funding acquisition (lead), investigation (lead), methodology (lead), project administration (lead), resources (lead), supervision (lead), writing – review and editing (lead).

## Conflicts of Interest

The authors declare no conflicts of interest.

## Supporting information


Data S1.

Data S2.

## Data Availability

The datasets used and/or analyzed during the current study are available from the corresponding author upon request.
