# Supplementary material for: Chidamide, a Histone Deacetylase Inhibitor, Combined With R‐GemOx in Relapsed/Refractory Diffuse Large B‐Cell Lymphoma (TRUST): A Multicenter, Single‐Arm, Phase 2 Trial
Source: Cancer Med. 2025 May 2;14(9):e70919. doi: 10.1002/cam4.70919 (PMC12046501; doi:10.1002/cam4.70919)
Supplement: Supplementary file 1 — Data S1. [file CAM4-14-e70919-s001.docx]

**Chidamide and R-GemOx(rituximab、gemcitabine plus oxaliplatin) regimen as salvage treatment for transplant-ineligible patients with relapsed /refractory diffuse large B-cell lymphoma: a multicenter, single arm, phase 2 study**

Version：v4.0

Date：August 25, 2019

### Summary

| Title | Chidamide and R-GemOx(rituximab, gemcitabine plus oxaliplatin) regimen as salvage treatment for transplant-ineligible patients with relapsed /refractory diffuse large B-cell lymphoma: a multicenter, single arm, phase 2 trial |
| --- | --- |
| Clinical study phase | Phase 2 |
| Population | Relapsed or refractory diffuse large B-cell lymphoma patients |
| Test drugs | 1. **Combined treatment period (induction phase) every 3 weeks for 6 cycles:**  - chidamide tablets, size: 5mg / tablet.   usage and dosage:  Oral administration, 20 mg (4 tablets), twice a week.   - The R-GemOx regimen   Rituximab，375mg/m^2^，d1， intravenous drip ；  Gemcitabine ，1000mg/m^2^，d2， intravenous drip ；  Oxaliplatin,1000mg/m^2^，d2， intravenous drip ；   1. **Maintenance treatment period (maintenance phase):**   Patients who achieved complete or partial response proceeded to chidamide monotherapy until disease progression or intolerable toxicity |
| Main criteria for inclusion /exclusion | **Main inclusion criteria:**  1. Patients were confirmed relapsed or refractory DLBCL  2. Patients were previously treated with anthracycline-based systemic chemotherapy.  3. Patients who are ineligible for autologous stem-cell transplantation (ASCT).  4. Patients with at least one assessable lesion.  5. Age between 18 and 75 years, both males and females.  6. ECOG Performance Status of 0-1.  7. Expected survival time of at least 3 months.  8. Hematopoietic function: absolute neutrophil count ≥1.5×10^9^/L, platelets ≥90×10^9^/L, hemoglobin ≥90 g/L; liver function: total bilirubin, ALT, and AST <1.5× upper limit of normal(ULN); patients with concomitant hepatitis B infection should receive effective antiviral therapy and have HBV-DNA <2000 IU/ml and ALT <2× ULN; renal function: Creatine <1.5× ULN and creatinine clearance rate ≥50 ml/min.  9. Normal cardiac and pulmonary function, without significant contraindications to chemotherapy.  10. No treatment with radiation therapy, chemotherapy, targeted therapy, cellular immunotherapy, or ASCT within 4 weeks prior to enrollment.  11. Voluntarily signed informed consent form before the screening for the trial.  **Main exclusion criteria:**  1. Double/Triple-hit lymphoma.  2. Patients previously treated with histone deacetylase inhibitors (HDACi).  3. Patients planning to undergo subsequent ASCT.  4. Involvement of the central nervous system.  5. Patients who received gemcitabine within the past 6 months.  6．Pregnant or breastfeeding women, or reproductive-age patients unwilling to use contraception.  7．Patients with a history of other tumors, excluding cured cervical cancer or basal cell carcinoma of the skin;  8. Patients who have undergone organ transplantation.  9. Patients who received symptomatic treatment for chemotherapy-induced bone marrow transplantation toxicity within 7 days prior to enrollment.  9．Patients with active bleeding.  11．Grade ≥2 peripheral neuropathy.  12．Patients with a clinically significant history of prolonged QT interval (males > 450ms, females > 470ms), ventricular tachycardia (VT), atrial fibrillation (AF), heart conduction block, myocardial infarction (MI), congestive heart failure (CHF), symptomatic coronary artery heart disease requiring medication.  14. Patients with mental disorders/unable to provide informed consent.  15. Patients using drugs or long-term alcohol abuse that could affect the evaluation of trial results.  16．Patients deemed unsuitable for participation in this trial by the investigator. |
| Endpoints | **The primary endpoint:**  Overall response rate (ORR)  **Secondary endpoints:**  Disease control rate (DCR)  Time to response (TTR)  Duration of response (DOR)  Progression-free survival (PFS)  Overall survival (OS)  Adverse events  **Exploratory endpoint**  The relationship between gene mutations and efficacy measures (ORR, PFS, and OS) |
| Trial procedure | 1. **Screening period**   After obtaining informed consent, eligible patients were screened for the trial by medical history, physical examination, laboratory examination, and tumor evaluation.   1. **The combined treatment period**   Patients took the medication according to the protocol and were followed at the prescribed time to evaluate the safety and efficacy.   1. Efficacy evaluation   Evaluation time: Tumor response was evaluated every 2 cycles (6 weeks). If the first efficacy assessment was SD and the researchers judged the benefit to the patients, patients can receive 2 more cycles of treatment.  Efficacy evaluation methods: Target lesions were evaluated using the same imaging methods as at baseline (neck / chest / abdominal / pelvic CT, MRI or whole-body PET-CT) and physical examination.  Efficacy evaluation criteria: Tumor response was evaluated in accordance with the 2014 Lugano criteria   1. **Maintenance treatment period**   Patients who achieved complete or partial response proceeded to chidamide monotherapy until disease progression or intolerable toxicity.  The dose of chidamide maintenance therapy was 20mg twice weekly or dosage during the combination treatment period.  Tumor response was evaluated every 8 weeks.   1. **Follow-up period**   Safety information is collected for all discontinued patients for at least 30 (up to +7) days after the last administration of study treatment, and both safety and efficacy information are collected for patients who discontinue study treatment without disease progression. Long-term follow-up was performed every 3 months in the first 2 years, every 6 months in 3-5 years, and every 12 months after 5 years. |
| Statistical analysis | **Sample size**  Sample size requirement was estimated using PASS version 15 software based on the following assumptions: 1) ORR with R-GemOx regimen at 44%; 2) ORR with CR-GemOx at 64% (20% absolute increase relative to R-GemOx); 3) 80% power and two-side significance level of 0.05. The calculation yielded 48 subjects. Assuming 10% drop-out, we planned to enroll 54 patients.  **Statistical analysis**  The baseline clinical characteristics, ORR, DCR, and safety were summarized by descriptive statistics. The two-sided 95% confidence interval (CI) for response was calculated by the Clopper-Pearson exact method. Survival outcomes of DOR, PFS, OS, and TTR were estimated by the Kaplan-Meier method, and compared using a log-rank test. Categorical variables were analyzed using the Chi-square test or Fisher’s exact test, as appropriate. p < 0.05 (2-sided) was considered statistically significant. |

###

### 1. Background

Diffuse large B-cell lymphoma (DLBCL) is the most common aggressive non-Hodgkin lymphoma (NHL) in adults, accounting for 30-40% of cases. According to the epidemiological data from GLOBOCAN 2018, there are 35,919 newly diagnosed DLBCL patients in China each year ^[1]^. DLBCL is a highly heterogeneous tumor, with different subtypes exhibiting distinct genetic alterations, clinical features, and treatment responses. The advent of immunochemotherapy has led to further improvements in the efficacy and prognosis of DLBCL. Currently, R-CHOP (rituximab, cyclophosphamide, doxorubicin, vincristine, prednisone) is the standard first-line treatment regimen, with more than 50% of DLBCL patients achieving cure and a 5-year survival rate of 60%-70% ^[2]^. However, 20-50% of patients still experience disease relapse or fail to achieve complete remission after treatment, referred to as relapsed/refractory DLBCL (R/R DLBCL) ^[3]^. This subgroup of patients has a poor prognosis.

Major guidelines recommend high-dose salvage chemotherapy followed by autologous stem-cell transplantation (ASCT) for chemotherapy-sensitive R/R DLBCL. However, for elderly patients or those with severe complications or resistance to treatment toxicity, who are unable to undergo ASCT, there is no standard treatment option. The current treatment strategies include participation in clinical trials, palliative radiotherapy, and salvage chemotherapy. Common salvage regimens include GemOx, GDP, bendamustine, lenalidomide, with or without rituximab combination ^[4-7]^.

Gemcitabine is a novel nucleoside analog, belonging to the class of antimetabolite antineoplastic drugs. It exhibits cell cycle specificity, primarily acting on S-phase cells, inhibiting tumor cell DNA synthesis, and inducing cell apoptosis. It has the advantages of fewer side effects and good tolerability. Several studies have confirmed the definite efficacy of gemcitabine in R/R DLBCL ^[4, 8]^. Oxaliplatin, compared to commonly used cisplatin, has lower renal and gastrointestinal toxicities. It has unique advantages in treating elderly patients with various comorbidities. The GemOx regimen (gemcitabine and oxaliplatin) has shown high efficacy and outstanding safety advantages in the treatment of RR DLBCL. Lopez et al. used the R-GemOx regimen to treat R/R DLBCL with an overall response rate (ORR) and complete response (CR) of 43% and 34%, respectively ^[5]^. The median overall survival (OS) was 9.1 months, and the 1-year OS and PFS rates were 41% and 29%, respectively. The most common grade 3 or higher toxicities were neutropenia/thrombocytopenia (43%) and neurotoxicity (7%). The study showed that R-GemOx is an efficient and relatively less toxic salvage regimen for RR DLBCL, especially for elderly patients unsuitable for ASCT ^[5]^. Corazzelli et al. conducted a clinical study using GemOx±R to treat transplant-ineligible RR B-cell lymphoma. The ORR and CR of the R-GemOx regimen were 78% and 50%, respectively, which showed that the GemOx±R regimen was well-tolerated, highly effective, and had a longer EFS ^[8]^. In a critical study enrolling 46 cases of R/R LBCL with 72% DLBCL, the ORR of R-GemOx regimen was 83%, with half of the patients achieving complete response (CR). The 2-year EFS and OS rates were 43% and 66%, respectively ^[9]^. Another study evaluating the use of the R-GemOx regimen in treating 49 elderly R/R DLBCL patients with a median age of 69 demonstrated an ORR of 61% and a CR of 44% after 4 cycles ^[10]^. These studies suggest that the R-GemOx regimen has a higher effectiveness and better safety for RR DLBCL patients unsuitable for transplantation.

Epigenetic factors such as histone acetyltransferases are essential during the differentiation and growth of hematopoietic stem cells into T, B lymphocytes, and other blood cells. Next-generation sequencing (NGS) has identified multiple binding sites for HDAC inhibitors in DLBCL patients ^[11]^. Furthermore, mutations in the histone acetylation regulatory factors CREBBP and EP300 have been found in 40% of DLBCL patient samples ^[12]^. Additionally, studies have discovered that 32% of DLBCL cases exhibit mutations in the epigenetic factor MLL2, and around 10% of DLBCL cases have MEF2B mutations, which are more common in germinal center-derived DLBCL and have interactions with CREBBP and EP300 ^[13]^. These mutations in epigenetic factors can lead to decreased acetylation levels, resulting in significant differences in "transcriptionally accessible regions" compared to normal cells, promoting tumor initiation and progression. Comprehensive evaluation of molecular events in the tumor progression process and their relationship with tumor prognosis and treatment efficacy is a recent hot topic in DLBCL research by NGS ^[14,15]^. As mentioned earlier, there are clearly defined epigenetic molecular events in DLBCL patients ^[12,13]^, and a phase II clinical study of panobinostat found that the degree of MEF2B gene mutation is most strongly correlated with ORR ^[16]^. Therefore, in addition to conventional radiological evaluation, the identification of high-risk populations in RR DLBCL through NGS and early detection of drug resistance or relapse can provide a basis for salvage treatment of RR DLBCL and support the precise treatment of chidamide.

Chidamide is an orally active HDAC inhibitor. In addition to good bioavailability upon oral administration, chidamide has higher selectivity and lower grade 3-4 toxicity than other HDAC inhibitors ^[17-19]^. In cultured DLBCL cells, chidamide and gemcitabine/oxaliplatin produced synergistic effects in inducing cell cycle arrest and promoting cell apoptosis ^[20, 21]^. The clinical efficacy of HDAC inhibitor was explored ^[16, 22, 23]^ . Additionally, the synergic effects between HDAC inhibitor and gemcitabine/platinum-based drugs were demonstrated in solid tumor ^[24,25]^. HDAC inhibitor could sensitizes rituximab-resistant DLBCL cells by upregulating CD20 expression ^[26, 27]^.

In previous clinical studies, chidamide has demonstrated efficacy in both monotherapy and combination therapy for B-cell lymphomas. In a phase I clinical study evaluating the treatment of R/R lymphomas, 27 evaluable patients received HDACi in combination with R and platinum or etoposide-based regimens, and 4 out of 6 R/R DLBCL patients achieved remission ^[28]^. In a clinical study of chidamide in combination with R-CHOP for the first-line treatment of elderly DLBCL, among 41 evaluable patients, the ORR and CR were 90.3% (37/41) and 85.4% (35/41), respectively. With a median follow-up of 18 months, 1-year PFS and OS rates of 92.1% and 94.7%, respectively ^[29]^. At the 2019 American T-cell Lymphoma Forum, N. Mehta-Shah et al. reported a phase I study included 6 DLBCL patients treated with romidepsin in combination with GemOx (gemcitabine 1000mg/m^2^ on day 1 plus oxaliplatin 100mg/m^2^ on day 1 every 3 weeks), with 2 patients achieving CR and DOR reaching 36.6 months and 8.5 months, respectively. The above studies demonstrate the good efficacy and safety of HDACi, including chidamide, in combination with chemotherapy for the treatment of DLBCL.

Based on these findings, we conducted a multicenter, single-arm, phase 2 trial (TRUST trial) to evaluate the efficacy and safety of chidamide in combination with R-GemOx (CR-GemOx) regimen for transplantation-ineligible R/R DLBCL patients.

### 2.Objectives

To evaluate the efficacy and safety of the combination of chidamide with R-GemOx regimen in the treatment of relapsed or refractory diffuse large B-cell lymphoma (DLBCL) patients who are ineligible for transplantation.

### 3.Study Design

This is a multicenter, single-arm, open-label phase 2 trial.

### 4.Study Population

##### 4.1 Sample Size

A total of 54 subjects are expected to be enrolled in this study at the participating research centers.

##### 4.2 Inclusion Criteria

The following criteria must be met for patient inclusion:

1. Patients were confirmed relapsed or refractory DLBCL
2. Patients were previously treated with anthracycline-based systemic chemotherapy.
3. Patients who are ineligible for autologous stem-cell transplantation (ASCT).
4. Patients with at least one assessable lesion.
5. Age between 18 and 75 years, both males and females.
6. ECOG Performance Status of 0-1.
7. Expected survival time of at least 3 months.
8. Hematopoietic function: absolute neutrophil count ≥1.5×109/L, platelets ≥90×109/L, hemoglobin ≥90 g/L; liver function: total bilirubin, ALT, and AST <1.5× upper limit of normal(ULN); patients with concomitant hepatitis B infection should receive effective antiviral therapy and have HBV-DNA <2000 IU/ml and ALT <2× ULN; renal function: Creatine <1.5× ULN and creatinine clearance rate ≥50 ml/min.
9. Normal cardiac and pulmonary function, without significant contraindications to chemotherapy.
10. No treatment with radiation therapy, chemotherapy, targeted therapy, cellular immunotherapy, or ASCT within 4 weeks prior to enrollment.
11. Voluntarily signed informed consent form before the screening for the trial.

##### 4.3 Exclusion Criteria

Patients meeting any of the following criteria are not eligible for inclusion:

1. Double/Triple-hit lymphoma.
2. Patients previously treated with histone deacetylase inhibitors (HDACi).
3. Patients planning to undergo subsequent ASCT.
4. Involvement of the central nervous system.
5. Patients who received gemcitabine within the past 6 months.
6. Pregnant or breastfeeding women, or reproductive-age patients unwilling to use contraception.
7. Patients with a history of other tumors, excluding cured cervical cancer or basal cell carcinoma of the skin;
8. Patients who have undergone organ transplantation.
9. Patients who received symptomatic treatment for chemotherapy-induced bone marrow transplantation toxicity within 7 days prior to enrollment.
10. Patients with active bleeding.
11. Grade ≥2 peripheral neuropathy.
12. Patients with a clinically significant history of prolonged QT interval (males > 450ms, females > 470ms), ventricular tachycardia (VT), atrial fibrillation (AF), heart conduction block, myocardial infarction (MI), congestive heart failure (CHF), symptomatic coronary artery heart disease requiring medication.
13. Patients with mental disorders/unable to provide informed consent.
14. Patients using drugs or long-term alcohol abuse that could affect the evaluation of trial results.
15. Patients deemed unsuitable for participation in this trial by the investigator.

### Treatment Plan

**5.1 Treatment Regimen**

The treatment consists of two phases: the induction phase and the maintenance phase.

**5.1.1 Induction Phase**

The administration schedule and dosages of each drug are as follows:

**Chidamide Tablets Dosage and Administration**: Oral administration, 20 mg (4 tablets) per dose, twice a week on days 0, 4, 7, 11, 14, and 18.

**R-GemOx Regimen Dosage and Administration**:

- Rituximab 375 mg/m^2^, day intravenous infusion. Prior to the infusion of rituximab, analgesics (e.g., acetaminophen) and antihistamines (e.g., diphenhydramine) should be administered premedication (30 to 60 minutes before the start of the infusion). Under aseptic conditions, the required dose of rituximab should be withdrawn and placed in an infusion bag containing sterile, non-pyrogenic 0.9% saline solution or 5% glucose solution, diluted to a concentration of 1 mg/ml. For the initial infusion, a recommended starting rate is 50 mg/h; after the first 60 minutes, the rate can be increased by 50 mg/h every 30 minutes up to a maximum rate of 400 mg/h. Subsequent infusions can be administered using a rapid intravenous infusion method, where 20% of the total dose of rituximab is infused over the first 30 minutes, and the remaining 80% is infused over the next 60 to 90 minutes.
- Gemcitabine 1000 mg/m^2^, day 2, intravenous infusion. Dissolve in 0.9% sodium chloride injection and infuse intravenously over 30 minutes.
- Oxaliplatin 100 mg/m^2^, day 2, intravenous infusion. Dilute in 250-500 ml of 5% glucose solution to achieve a concentration of 0.2 mg/ml or higher, and infuse intravenously over 2-6 hours.

**5.1.2 Maintenance Phase**

Patients who achieved complete or partial response proceeded to chidamide monotherapy until disease progression or intolerable toxicity.

The dose of chidamide maintenance therapy was 20mg twice weekly or dosage during the combination treatment period.

**5.2 Dosage Adjustment**

1. In the case of grade 4 hematologic toxicity other than neutropenia or grade 3 thrombocytopenia with significant bleeding tendency, the investigator decided to adjust the dosage level of chidamide, gemcitabine, and/or oxaliplatin based on the patient's general condition and comorbidities, reducing each dosage level by one level.

2. In the event of grade≥3 non-hematologic toxicity, considering the correlation between non-hematologic adverse events and the investigational drugs, if deemed related, the corresponding drug was temporarily discontinued until the non-hematologic toxicity improved to grade ≤1 or the pre-treatment level. When resuming the drug, the dosage of the corresponding drug was reduced by one level. If deemed unrelated, treatment was continued.

3. During the course of treatment, if the dosage of chidamide was reduced to 10 mg or the dosage of any drug in the chemotherapy regimen was reduced to 50% of the initial dosage and tolerance issues persisted, treatment should be discontinued.

4. The dosage adjustment levels for the drugs are shown in the table below.

| Dosage Level | Chidamide | Gemcitabine | Oxaliplatin |
| --- | --- | --- | --- |
| Dosage Level 1 | 20mg | 1000mg/m^2^ | 100mg/m^2^ |
| Dosage Level 2 | 15mg | 750mg/m^2^ | 75mg/m^2^ |
| Dosage Level 3 | 10mg | 500mg/m^2^ | 50mg/m^2^ |

5.4 Combination Therapy

During the study period, symptomatic treatment may be administered for adverse events based on the protocol or the medical judgment of the investigator. No other anti-tumor treatments should be administered during the trial to avoid interference with the evaluation of the investigational drug's efficacy.

The specific provisions are as follows:

1. Throughout the entire trial, patients are not allowed to receive any other anti-tumor measures, including chemotherapy, targeted therapy, radiation therapy, hematopoietic stem cell transplantation, and anti-tumor traditional Chinese medicine.
2. The investigator may provide appropriate supportive therapy after evaluating the relationship between adverse events and the investigational drug. The initiation and duration of supportive treatment should be recorded in the original records. These treatments include antiemetics, antidiarrheals, antipyretics, antiallergics, diabetes treatment, use of antihypotensive drugs, analgesics, antibiotics, and others (such as the use of blood products).
3. Symptomatic treatment should be provided when hematologic toxicity of grade 3 , and it should be documented as part of combination therapy.
4. Symptomatic treatment should be provided for non-hematologic toxicity of grade 2, and it should be recorded as part of combination therapy.
5. Maintenance treatment should be provided for underlying diseases.
6. The use of other investigational drugs during the trial is prohibited.
7. All information regarding the concomitant medication during the study (generic name of the drug, purpose of administration, dosage, administration time, etc.) must be accurately documented in the source data.

##### 6.1 Evaluation of Response

6.1.1 Evaluation Interval for Efficacy

The evaluation intervals for efficacy in patients are as follows:

- Tumor response was performed during the screening period.
- Tumor response was performed every 6 weeks during the combination therapy period, and every 8 weeks during the maintenance therapy period.

6.1.2 Evaluation Methods

Evaluation of lymph nodes and organ lesions is performed using imaging techniques such as computed tomography (CT), magnetic resonance imaging (MRI), or whole-body positron emission tomography-computed tomography (PET-CT), as well as clinical examinations, bone marrow aspiration, and biopsy. The imaging methods used at all follow-up time points must be consistent.

6.1.3 Evaluation Criteria

Tumor response was evaluated in accordance with the 2014 Lugano criteria

6.2 Safety Evaluation

6.2.1 Safety Evaluation Methods

Adverse events (AEs) were recorded at every visit and graded using the National Cancer Institute Common Terminology Criteria for Adverse Events version 5.0 (NCI-CTCAE 5.0). Serious adverse events (SAEs) were defined as AEs that led to or prolonged hospitalization, life-threatening event, death, or permanent disability.

6.2.2 Safety Observation Indicators

6.2.2.1 Physical Examination

The physical examination was conducted according to the schedule in the protocol. A comprehensive physical examination was performed, including assessments of general condition, skin, head and neck, chest, abdomen, back, extremities, and basic neurological system. The information from the physical examination was recorded in the patient's original records.

6.2.2.2 Vital Signs

Vital signs were measured according to the schedule in the protocol. Each visit involved collecting the patient's height (during the screening period only), weight, body temperature, blood pressure, pulse rate, and respiratory rate.

6.2.2.3 ECOG Performance Status

ECOG performance status assessment was conducted according to the schedule in the protocol. The assessment criteria are provided in Appendix 4.

6.2.2.4 Laboratory Tests

The following laboratory tests were performed:

Hematology: including white blood cell count and differential count or percentage, hemoglobin, red blood cell count, and platelet count

Blood chemistry: including ALT, AST, GGT, ALB, TBIL, DBIL, BUN, Cr, Mg2+, Ca2+, K+, Na+, Cl-, fasting blood glucose, and LDH

Urinalysis: including white blood cells, urinary protein, red blood cells, and urinary glucose

Electrocardiogram: calculating and recording the QTc interval

6.2.3 Adverse Events (AE)

AE refers to any unfavorable medical occurrence that happens to a clinical trial subject after signing the informed consent form. In this trial, AE encompasses any adverse medical event occurring within 90 days after the last administration of the study drug, regardless of causal relationship. Baseline abnormalities in physical examination or laboratory tests, which worsen in severity after drug treatment, are also considered as AE.

The researchers should thoroughly document any AE experienced by the patients, including a description of the AE and all related symptoms, occurrence time, severity, duration, measures taken, and final outcome and resolution.

6.2.3.1 Criteria for Determining the Severity of Adverse Events

Refer to the grading criteria for drug adverse reactions in NCI-CTC AE5.0 version. If adverse events occur that are not listed in the table, the following criteria can be used:

Grade 1: Mild, no clinical symptoms or mild clinical symptoms; only clinical or laboratory abnormalities without the need for treatment.

Grade 2: Moderate, requiring minimal, local, or non-invasive treatment; limitations in activities of daily living using age-appropriate tools, such as cooking, shopping, making phone calls, counting money, etc.

Grade 3: Severe illness or medically significant symptoms that are not immediately life-threatening; resulting in hospitalization or prolonged hospital stay; leading to disability; limitations in activities of daily living. Activities of daily living include bathing, dressing, undressing, eating, using the toilet, taking medication, etc., except when confined to bed.

Grade 4: Life-threatening, requiring urgent treatment.

Grade 5: AE-related death.

6.2.4 Serious Adverse Events (SAE)

6.2.4.1 Definition of Serious Adverse Events

SAE refers to medical events that occur during the clinical trial process and require hospitalization or result in prolonged hospital stay, disability, impairment of work capacity, life-threatening situations, death, congenital anomalies, etc. It includes the following unexpected medical events:

- Events resulting in death
- Life-threatening events (defined as patients being at risk of death at the time of the event)
- Events requiring hospitalization or prolongation of existing hospitalization
- Events causing permanent or severe disability/dysfunction
- Congenital anomalies or birth defects
- Other significant medical events that require intervention to prevent permanent damage or harm.

6.2.4.2 Administration of Other Antitumor Treatments

For non-fatal adverse events (AEs), the reporting period extends until the initiation of new antitumor treatment. If death occurs after the completion of the study treatment but within the SAE reporting period, it must be reported promptly, regardless of whether the patient received other treatments.

6.2.4.3 Hospitalization

Adverse events that lead to hospitalization or an extended hospital stay during a clinical study are considered SAEs, excluding non-medical hospitalization. Hospitalization or an extended stay unrelated to AE worsening itself is not an SAE. For example:

- Admission due to pre-existing conditions without the occurrence of new AEs or exacerbation of pre-existing diseases (e.g., for the investigation of pre-existing laboratory abnormalities since the start of the trial).
- Hospitalization for administrative reasons (e.g., routine annual check-ups).
- Protocol-specified hospitalization during the clinical trial (e.g., required procedures according to the trial protocol).
- selective hospitalization unrelated to AE worsening (e.g., selective cosmetic surgery).
- Pre-scheduled treatment or surgery should be recorded in the entire trial protocol and/or the patient's baseline data.
- Hospitalization solely for blood product use.

Diagnostic or therapeutic invasive procedures (e.g., surgery) and non-invasive interventions should not be reported as AEs. However, if the disease condition that led to such procedures meets the definition of an AE, it should be reported. For example, acute appendicitis occurring during the AE reporting period should be reported as an AE, and the subsequent appendectomy should be recorded as the treatment method for that AE.

6.2.4.4 Reporting of Serious Adverse Events

The reporting of Serious Adverse Events (SAEs) should begin from the date of patient's informed consent signature and continue for a period of 90 calendar days (including 90 days) after the last administration of the investigational drug. During the trial, if an SAE occurs, whether it is the initial report or a follow-up report, the investigator must promptly complete the "Clinical Trial Serious Adverse Event (SAE) Report Form" with their signature and date. Within 24 hours of becoming aware of the SAE, the investigator must immediately notify the clinical monitor, report to the research unit's ethics committee, the China Food and Drug Administration (CFDA), the food and drug regulatory authority of the investigator's region (province or city), and the medical administration department of the health authority.

During the continuation of drug supply after the completion of the study, any SAEs that occur must be reported to the sponsor within 24 hours of the investigator becoming aware of them. All information regarding SAEs should be recorded in the SAE form. SAEs occurring within 90 days after the last drug administration during the continuation of drug supply period must be reported. SAEs occurring 90 days after the last drug administration generally do not require reporting unless there is suspicion of a relationship with the investigational drug.

SAEs should be documented in detail, including symptoms, severity, occurrence and handling time, measures taken, follow-up time and method, and outcomes. If the investigator believes that an SAE is unrelated to the investigational drug but potentially related to the study conditions (such as discontinuation of previous treatment or concomitant events during the trial), this relationship should be clearly explained in the narrative section of the SAE page in the medical report form. If there is a change in the intensity of an ongoing SAE or its relationship with the investigational drug, the SAE follow-up report should be promptly submitted to the sponsor. All SAEs should be followed up until recovery or stabilization.

6.2.4.5 Pregnancy

If female subjects become pregnant, they will be discontinued from the trial. If the partners of male subjects become pregnant, the clinical trial will continue. The researchers are required to follow up on the pregnancy outcomes for one month after delivery. If negative pregnancy outcomes (stillbirth, spontaneous abortion, fetal abnormalities) are considered serious adverse events (SAEs), they must be reported within the specified time frame according to the SAE reporting requirements. If patients meet the criteria for both SAEs, an SAE report form should be completed and reported as per the SAE requirements.

6.2.4.6 Follow-up of Adverse Events/Serious Adverse Events

All SAEs and drug-related adverse events (AEs) should be followed up until they resolve, improve to baseline levels or ≤ Grade 1, reach a stable state, or receive a reasonable explanation (such as loss to follow-up, death). Researchers should inquire about any AE/SAE occurrences since the last visit and whether there are any new AE/SAEs at each visit, documenting relevant updates including outcomes.

### Statistical Methods and Data Analysis

##### 7.1 Sample Size Estimation

Sample size requirement was estimated using PASS version 15 software based on the following assumptions: 1) ORR with R-GemOx regimen at 44%; 2) ORR with CR-GemOx at 64% (20% absolute increase relative to R-GemOx); 3) 80% power and two-side significance level of 0.05. The calculation yielded 48 subjects. Assuming 10% drop-out, we planned to enroll 54 patients.

**7.2Data Analysis**

The baseline clinical characteristics, ORR, DCR, and safety were summarized by descriptive statistics. The two-sided 95% confidence interval (CI) for response was calculated by the Clopper-Pearson exact method. Survival outcomes of DOR, PFS, OS, and TTR were estimated by the Kaplan-Meier method, and compared using a log-rank test. Categorical variables were analyzed using the Chi-square test or Fisher’s exact test, as appropriate. p < 0.05 (2-sided) was considered statistically significant.

.

### 8.Informed Consent, Data Protection, and Trial Records

The conduct of this trial complies with the requirements of the "Good Clinical Practice for Drug Clinical Trials" and the "Helsinki Declaration" in China. Similar to routine medical practice, researchers in this trial bear the responsibility of diagnosing and treating patients.

In the event of any urgent safety issues during the trial, measures must be taken to prevent harm to the patients or any serious deviations from the trial protocol, Good Clinical Practice (GCP), or relevant provisions of the Helsinki Declaration. It is the responsibility of the researchers to promptly inform the study sponsor of such occurrences.

##### 8.1 Ethical Approval and Informed Consent

Prior to the commencement of the trial, the trial protocol, informed consent form, and other trial documents must receive approval from the responsible unit's ethics committee for this clinical trial. Researchers are obligated to report the progress of the trial and any serious adverse events that occur during the trial to the ethics committee. Any modifications to the trial protocol or informed consent form require submission to the ethics committee for review and approval.

Before patients are enrolled in this trial, researchers must provide comprehensive information to the subjects (or their legally authorized representatives in special circumstances) regarding the purpose, procedures, and potential risks and benefits of the clinical trial. Subjects should be made aware of their right to withdraw from the trial at any time. Prior to enrollment, each subject (or their legally authorized representative in special circumstances) must sign the informed consent form. The research physician is responsible for obtaining the signed informed consent form, including the names, dates, and contact information of both the physician and the subject, before the subject enters the study. The original copy is kept as a trial document at the institution, and a copy is provided to the subject for retention.

Any changes to the informed consent form must be documented and submitted to the ethics committee for review and approval. The formally implemented new version of the informed consent form must also be archived at the institution. Each patient or independent witness affected by the modifications to the informed consent form, as well as those involved in the informed consent form discussions, must sign and date the new version of the informed consent form. The original copy is kept as a trial document at the institution, and a copy is provided to the subject for retention.

##### 8.2 Confidentiality of Participant Information

The confidentiality of participant information is strictly enforced by the researchers and all individuals involved in the study. The research protocol, documents, data, and all other generated information are kept strictly confidential. Without prior written approval from the research institution, no relevant research or data information shall be disclosed to any unauthorized third party. Other authorized representatives of the researchers, the Institutional Review Board (IRB), or regulatory authorities, as well as representatives from the pharmaceutical company providing investigational drugs for the study, may inspect all files and records that researchers are required to maintain. These include, but are not limited to, medical records and participants' drug administration records. The research center should allow access to these records. Participant contact information will be securely stored at the research center and used only for internal purposes during the research process. Participant research data collected for statistical analysis and scientific reporting will be uploaded and stored at Sun Yat-sen University Cancer Center. This data should not include participant contact information or identifying information. At the end of the study, all identifying information in the research databases will be eliminated and archived at Sun Yat-sen University Cancer Center.

##### 8.3 Data Quality Assurance

The trial center should establish an internal quality assurance system to rigorously monitor and control the trial quality at the center. The center should ensure the accuracy and reliability of laboratory test results and establish uniform criteria for determining abnormal values. All relevant laboratory testing data in the trial should be promptly and accurately recorded in the Case Report Form (CRF), and the original reports or their copies should be attached to the research medical records. Any abnormal data should be verified, and necessary explanations should be provided by the respective researchers. If necessary, tracking and follow-up should be conducted until the data returns to normal or stabilizes.

##### 8.4 Deviations from the Protocol

The researchers/assistant researchers should maintain detailed records of any deviations from the protocol that occur during the trial. These records should be submitted to the principal investigator of the center and the ethics committee, along with an explanation of whether the deviations were made to eliminate potential harm to the patients or due to other unavoidable medical reasons.

### reference

1. Bray F, Ferlay J, Soerjomataram I, Siegel RL, Torre LA, Jemal A: Global cancer statistics 2018: GLOBOCAN estimates of incidence and mortality worldwide for 36 cancers in 185 countries. CA Cancer J Clin 2018.

2. Feugier P, Van Hoof A, Sebban C, Solal-Celigny P, Bouabdallah R, Ferme C, Christian B, Lepage E, Tilly H, Morschhauser F et al: Long-term results of the R-CHOP study in the treatment of elderly patients with diffuse large B-cell lymphoma: a study by the Groupe d'Etude des Lymphomes de l'Adulte. J Clin Oncol 2005, 23(18):4117-4126.

3. Sehn LH, Berry B, Chhanabhai M, Fitzgerald C, Gill K, Hoskins P, Klasa R, Savage KJ, Shenkier T, Sutherland J et al: The revised International Prognostic Index (R-IPI) is a better predictor of outcome than the standard IPI for patients with diffuse large B-cell lymphoma treated with R-CHOP. Blood 2007, 109(5):1857-1861.

4. Crump M, Baetz T, Couban S, Belch A, Marcellus D, Howson-Jan K, Imrie K, Myers R, Adams G, Ding K et al: Gemcitabine, dexamethasone, and cisplatin in patients with recurrent or refractory aggressive histology B-cell non-Hodgkin lymphoma: a Phase II study by the National Cancer Institute of Canada Clinical Trials Group (NCIC-CTG). Cancer 2004, 101(8):1835-1842.

5. Lopez A, Gutierrez A, Palacios A, Blancas I, Navarrete M, Morey M, Perello A, Alarcon J, Martinez J, Rodriguez J: GEMOX-R regimen is a highly effective salvage regimen in patients with refractory/relapsing diffuse large-cell lymphoma: a phase II study. Eur J Haematol 2008, 80(2):127-132.

6. Ohmachi K, Niitsu N, Uchida T, Kim SJ, Ando K, Takahashi N, Takahashi N, Uike N, Eom HS, Chae YS et al: Multicenter phase II study of bendamustine plus rituximab in patients with relapsed or refractory diffuse large B-cell lymphoma. J Clin Oncol 2013, 31(17):2103-2109.

7. Wang M, Fowler N, Wagner-Bartak N, Feng L, Romaguera J, Neelapu SS, Hagemeister F, Fanale M, Oki Y, Pro B et al: Oral lenalidomide with rituximab in relapsed or refractory diffuse large cell, follicular and transformed lymphoma: a phase II clinical trial. Leukemia 2013, 27(9):1902-1909.

8. Corazzelli G, Capobianco G, Arcamone M, Ballerini PF, Iannitto E, Russo F, Frigeri F, Becchimanzi C, Marcacci G, De ChiaraA et al: Long-term results of gemcitabine plus oxaliplatin with and without rituximab as salvage treatment for transplant-ineligible patients with refractory/relapsing B-cell lymphoma. Cancer Chemother Pharmacol 2009, 64(5):907-916.

9. El Gnaoui T, Dupuis J, Belhadj K, Jais JP, Rahmouni A, Copie-Bergman C, GaillardI, Divine M, Tabah-Fisch I, Reyes F et al: Rituximab, gemcitabine and oxaliplatin:an effective salvage regimen for patients with relapsed or refractory B-cell lymphoma not candidates for high-dose therapy. Ann Oncol 2007, 18(8):1363-1368.

10. Mounier N, El Gnaoui T, Tilly H, Canioni D, Sebban C, asasnovas RO, Delarue R, Sonet A, Beaussart P, Petrella T et al: Rituximab plus gemcitabine and oxaliplatin in patients with refractory/relapsed diffuse large B-cell lymphoma who are not candidates for high-dose therapy. A phase II Lymphoma Study Association trial. Haematologica 2013, 98(11):1726-1731.

11. Dubois S, Viailly PJ, Bohers E, Bertrand P, Ruminy P, Marchand V, Maingonnat C, Mareschal S, Picquenot JM, Penther D et al: Biological and clinical relevance of associated genomic alterations in MYD88 L265P and non-L265P-mutated diffuse large B-cell lymphoma: analysis of 361 cases. Clin Cancer Res 2017, 23(9):2232-2244.

12. Pasqualucci L, Dominguez-Sola D, Chiarenza A, Fabbri G, Grunn A, Trifonov V, Kasper LH, Lerach S, Tang H, Ma J et al: Inactivating mutations of acetyltransferase genes in B-cell lymphoma. Nature 2011, 471(7337):189-195.

13. Morin RD, Mendez-Lago M, Mungall AJ, Goya R, Mungall KL, Corbett RD, Johnson NA, Severson TM, Chiu R, Field M et al: Frequent mutation of histone-modifying genes in non-Hodgkin lymphoma. Nature 2011, 476(7360):298-303.

14. Schmitz R, Wright GW, Huang DW, Johnson CA, Phelan JD, Wang JQ, Roulland S, Kasbekar M, Young RM, Shaffer AL et al: Genetics and pathogenesis of diffuse large B-cell lymphoma. N Engl J Med 2018, 378(15):1396-1407.

15. Reddy A, Zhang J, Davis NS, Moffitt AB, Love CL, Waldrop A, Leppa S, Pasanen A, Meriranta L, Karjalainen-Lindsberg ML et al: Genetic and functional drivers of diffuse large B cell lymphoma. Cell 2017, 171(2):481-494 e415.

16. Assouline SE, Nielsen TH, Yu S, Alcaide M, Chong L, MacDonald D, Tosikyan A, Kukreti V, Kezouh A, Petrogiannis-Haliotis T et al: Phase 2 study of panobinostat with or without rituximab in relapsed diffuse large B-cell lymphoma. Blood 2016, 128(2):185-194.

17. Xu Y, Zhang P, Liu Y: Chidamide tablets: HDAC inhibition to treat lymphoma. Drugs Today (Barc) 2017, 53(3):167-176.

18. Apuri S, Sokol L: An overview of investigational histone deacetylase inhibitors (HDACis) for the treatment of non-Hodgkin's lymphoma. Expert Opin Investig Drugs 2016, 25(6):687-696.

19. Suresh PS, Devaraj VC, Srinivas NR, Mullangi R: Review of bioanalytical assays for the quantitation of various HDAC inhibitors such as vorinostat, belinostat, panobinostat, romidepsin and chidamine. Biomed Chromatogr 2017, 31(1).

20. Cai Y, Cui W, Chen W, Wei P, Chi Y, Zhang P, Bi R, Zhou X: The effects of a histone deacetylase inhibitor on biological behavior of diffuse large B-cell lymphoma cell lines and insights into the underlying mechanisms. Cancer Cell Int 2013, 13(1):57.

21. Xue K, Gu JJ, Zhang Q, Mavis C, Hernandez-Ilizaliturri FJ, Czuczman MS, Guo Y: Vorinostat, a histone deacetylase (HDAC) inhibitor, promotes cell cycle arrest and re-sensitizes rituximab- and chemo-resistant lymphoma cells to chemotherapy agents. J Cancer Res Clin Oncol 2016, 142(2):379-387.

22. Batlevi CL, Crump M, Andreadis C, Rizzieri D, Assouline SE, Fox S, van der Jagt RHC, Copeland A, Potvin D, Chao R et al: A phase 2 study of mocetinostat, a histone deacetylase inhibitor, in relapsed or refractory lymphoma. Br J Haematol 2017, 178(3):434-441.

23.Huiqiang Huang et al. Chidamide, oral subtype-selective histone deacetylase inhibitor (HDACI) monotherapy was effective on the patients with relapsed or refractory extranodal natural killer (NK)/T-cell lymphoma.2017 ASH.

24. Qiao Z, Ren S, Li W, Wang X, He M, Guo Y, Sun L, He Y, Ge Y, Yu Q: Chidamide, a novel histone deacetylase inhibitor, synergistically enhances gemcitabine cytotoxicity in pancreatic cancer cells. Biochem Biophys Res Commun 2013, 434(1):95-101.

25. Zhou Y, Pan DS, Shan S, Zhu JZ, Zhang K, Yue XP, Nie LP, Wan J, Lu XP, Zhang W et al: Non-toxic dose chidamide synergistically enhances platinum-induced DNA damage responses and apoptosis in Non-Small-Cell lung cancer cells. Biomed Pharmacother 2014, 68(4):483-491.

26. Shimizu R, Kikuchi J, Wada T, Ozawa K, Kano Y, Furukawa Y: HDAC inhibitors augment cytotoxic activity of rituximab by upregulating CD20 expression on lymphoma cells. Leukemia 2010, 24(10):1760-1768.

27. Frys S, Simons Z, Hu Q, Barth MJ, Gu JJ, Mavis C, Skitzki J, Song L, Czuczman MS, Hernandez-Ilizaliturri FJ: Entinostat, a novel histone deacetylase inhibitor is active in B-cell lymphoma and enhances the anti-tumour activity of rituximab and chemotherapy agents. Br J Haematol 2015, 169(4):506-519.

28. Budde LE, Zhang MM, Shustov AR, Pagel JM, Gooley TA, Oliveira GR, Chen TL, Knudsen NL, Roden JE, Kammerer BE et al: A phase I study of pulse high-dose vorinostat (V) plus rituximab (R), ifosphamide, carboplatin, and etoposide (ICE) in patients with relapsed lymphoma. Br J Haematol 2013, 161(2):183- 191.

29. Muchen Zhang et al. Chidamide plus R-CHOP21 in elderly patients with newly diagnosed diffuse large B-cell lymphoma: Results of a phase II study. 2018 ASH. Paper No:2968.
