# Supplementary material for: Chidamide, a Histone Deacetylase Inhibitor, Combined With R‐GemOx in Relapsed/Refractory Diffuse Large B‐Cell Lymphoma (TRUST): A Multicenter, Single‐Arm, Phase 2 Trial
Source: Cancer Med. 2025 May 2;14(9):e70919. doi: 10.1002/cam4.70919 (PMC12046501; doi:10.1002/cam4.70919)
Supplement: Supplementary file 2 — Data S2. [file CAM4-14-e70919-s002.doc]

**Supplementary materials**

**Contents**

[Supplementary methods 2](#__RefHeading___Toc194597802)

[Supplementary table 1. Subsequent treatment after the failure of CR-GemOx 3](#__RefHeading___Toc194597803)

[Figure S1. Forest plot showing subgroup analyses of ORR in efficacy-evaluable population. 4](#__RefHeading___Toc194597804)

[Figure S2. Subgroup analyses of PFS. 5](#__RefHeading___Toc194597805)

[Figure S3. Subgroup analyses of PFS. 6](#__RefHeading___Toc194597806)

[Figure S4. Subgroup analyses of OS. 7](#__RefHeading___Toc194597807)

[Figure S5. Subgroup analyses of OS. 8](#__RefHeading___Toc194597808)

[Figure S6. Transcriptomic analyses. 10](#__RefHeading___Toc194597809)

**Supplementary methods**

**(1) Dose-adjustment schema**

Dose delay (no more than 14 days) and reduction by 25% were permitted in case of grade 4 neutropenia or thrombocytopenia, grade 3 or more febrile neutropenia, grade 3 or more thrombocytopenia with bleeding events, or grade 3 or more non-hematological treatment-emergent adverse events (TEAEs). Treatment was discontinued permanently if the toxicity did not resolve after a 2-week delay. A maximum of 2 dose reductions were permitted.

**(2) Whole-exome sequencing (WES)**

DNA was extracted from formalin-fixed paraffin-embedded (FFPE) tissues using the QIAamp DNA FFPE tissue Kit (QIAGEN, GmBH, Germany). The concentration and quality of purified DNA were determined using Qubit 2.0 Fluorometer (Invitrogen) and 1% agarose gel electrophoresis, respectively. DNA fragmentation, end-repair, a-tailing, adapter ligation, and polymerase chain reaction (PCR) were conducted using SureSelectXT Target Enrichment System for Illumina Paired-End Sequencing Library (Agilent Technologies, CA, USA). Product quality and size were determined using Agilent 2100 Bioanalyzer High Sensitivity DNA Assay. TruSeq PE Cluster Kit (Illumina) was used for cluster generation in an Illumina cBOT instrument. Pair-end sequencing was performed using an Illumina HiSeq X instrument (Illumina).

**(3) RNA sequencing (RNAseq)**

RNA was extracted from FFPE tissues followed by library preparation using the VAHTS Universal V6 RNA-seq Library Prep Kit for Illumina. Agilent 4200 bioanalyzer was used to evaluate the concentration and size distribution of cDNA library before sequencing with an Illumina novaseq6000. The raw reads were filtered by Seqtk before mapping to genome using Hisat2 (version:2.0.4). Gene fragments were counted using stringtie (v1.3.3b) followed by TMM (trimmed mean of M values) normalization. Differentially expressed genes (DEGs) were defined by fold-change >1.75 and false discovery rate (FDR) above the threshold (Q< 0.05).

**Supplementary table 1. Subsequent treatment after the failure of CR-GemOx**

| **Patients’ number** | **Subsequent treatment** |
| --- | --- |
| P10 | PI3Kδ inhibitor + gemcitabine + oxaliplatin; CD47 monoclonal antibody; bispecific antibody |
| P14 | rituximab + lenalidomide |
| P18 | rituximab + ifosfamide + etoposide + lenalidomide |
| P22 | lenalidomide; bendamustine; anti-PD1 antibody + albumin-bound paclitaxel; BTK inhibitor + lenalidomide; venetoclax + ifosfamide + etoposide |
| P23 | CAR-T therapy; rituximab + cyclophosphamide + vincristine + doxorubicin + prednisone + BTK inhibitor |
| P24 | anti-PD1 antibody + ifosfamide + carboplatin + etoposide |
| P29 | ifosfamide + etoposide + lenalidomide; bendamustine |
| P32 | rituximab + cyclophosphamide + vincristine + doxorubicin + prednisone; anti-PD1 antibody + lenalidomide |
| P35 | rituximab + bendamustine |
| P40 | rituximab + ifosfamide + carboplatin + etoposide |
| P42 | BTK inhibitor; rituximab + lenalidomide; anti-PD1 antibody + albumin-bound paclitaxel + BTK inhibitor |
| P47 | BTK inhibitor; CD20 antibody-drug conjugate |
| P48 | BTK inhibitor |
| P51 | CD20 antibody-drug conjugate |
| P52 | anti-PD1 antibody + albumin-bound paclitaxel + BTK inhibitor; bispecific antibody |
| P56 | obinutuzumab + DA-EPOCH; polatuzumab + obinutuzumab + bendamustine; BTK inhibitor + CAR-T therapy |
| P57 | anti-PD1 antibody + albumin-bound paclitaxel + BTK inhibitor; bispecific antibody |
| P58 | BTK inhibitor; anti-PD1 antibody + albumin-bound paclitaxel + BTK inhibitor |


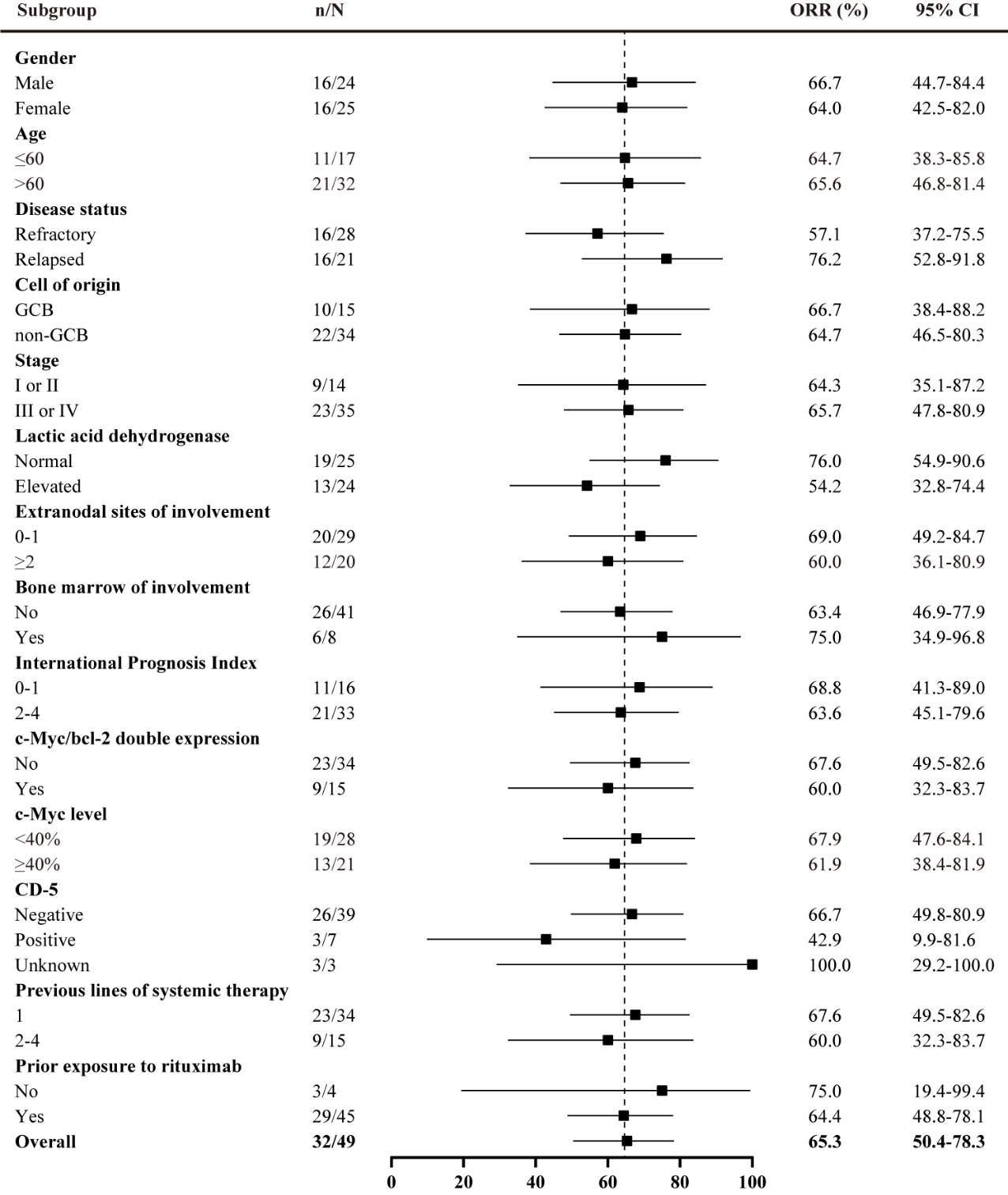


**Figure S1.** **Forest plot showing subgroup analyses of ORR in efficacy-evaluable population.**

Abbreviations: CI, confidence interval; GCB, germinal center B cell; ORR, objective response rate.

**
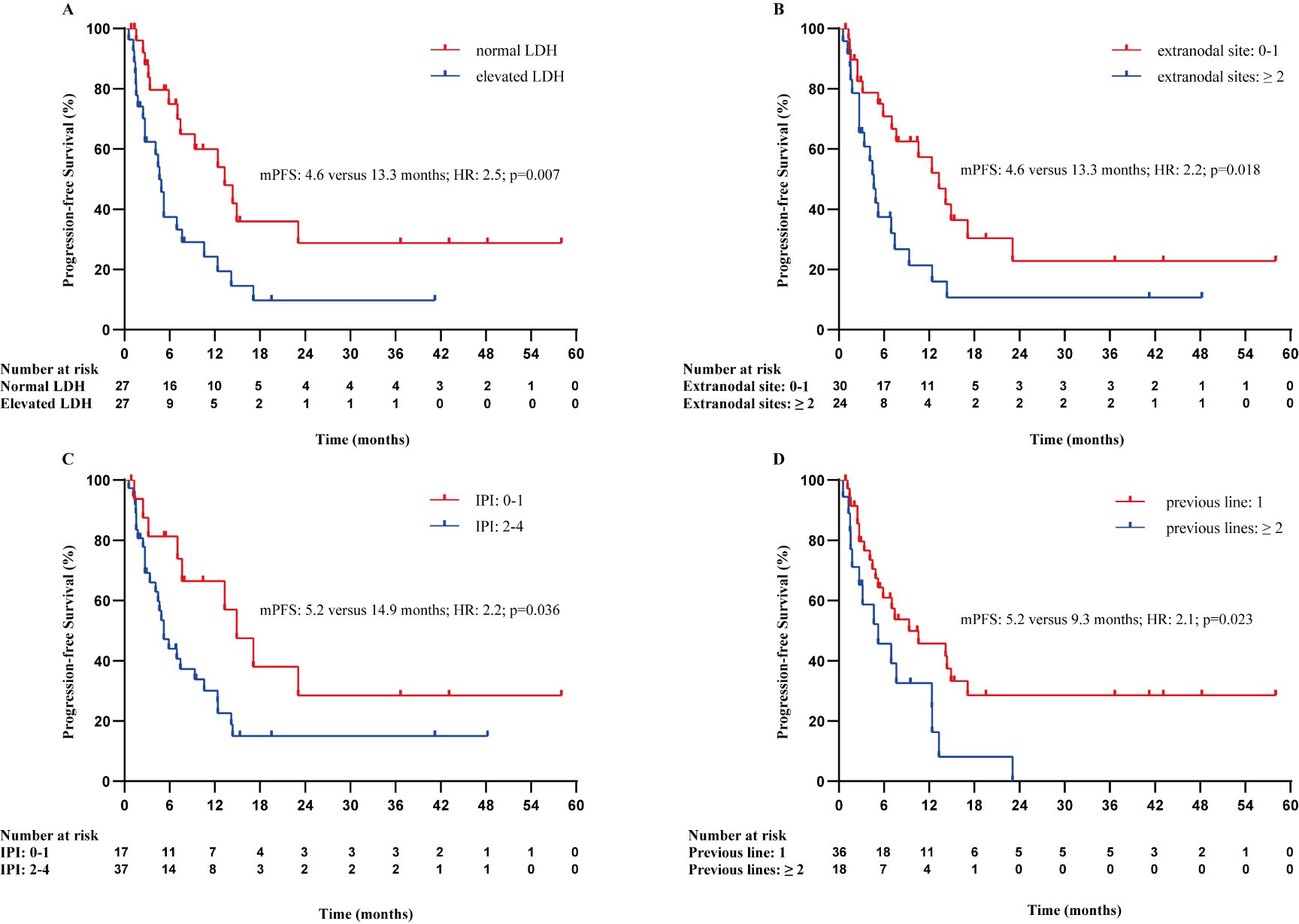
**

**Figure S2. Subgroup analyses of PFS**.

(A) PFS of the patients with normal versus elevated LDH. (B) PFS of the patients with involvement of 0-1 versus ≥ 2 extranodal sites. (C) PFS of the patients with 0-1 versus 2-4 IPI. (D) PFS of the patients with 1 versus ≥ 2 previous lines of therapy.

Abbreviations: HR, hazard ratio; IPI, International Prognostic Index; LDH, lactate dehydrogenase; mPFS, median progression-free survival.


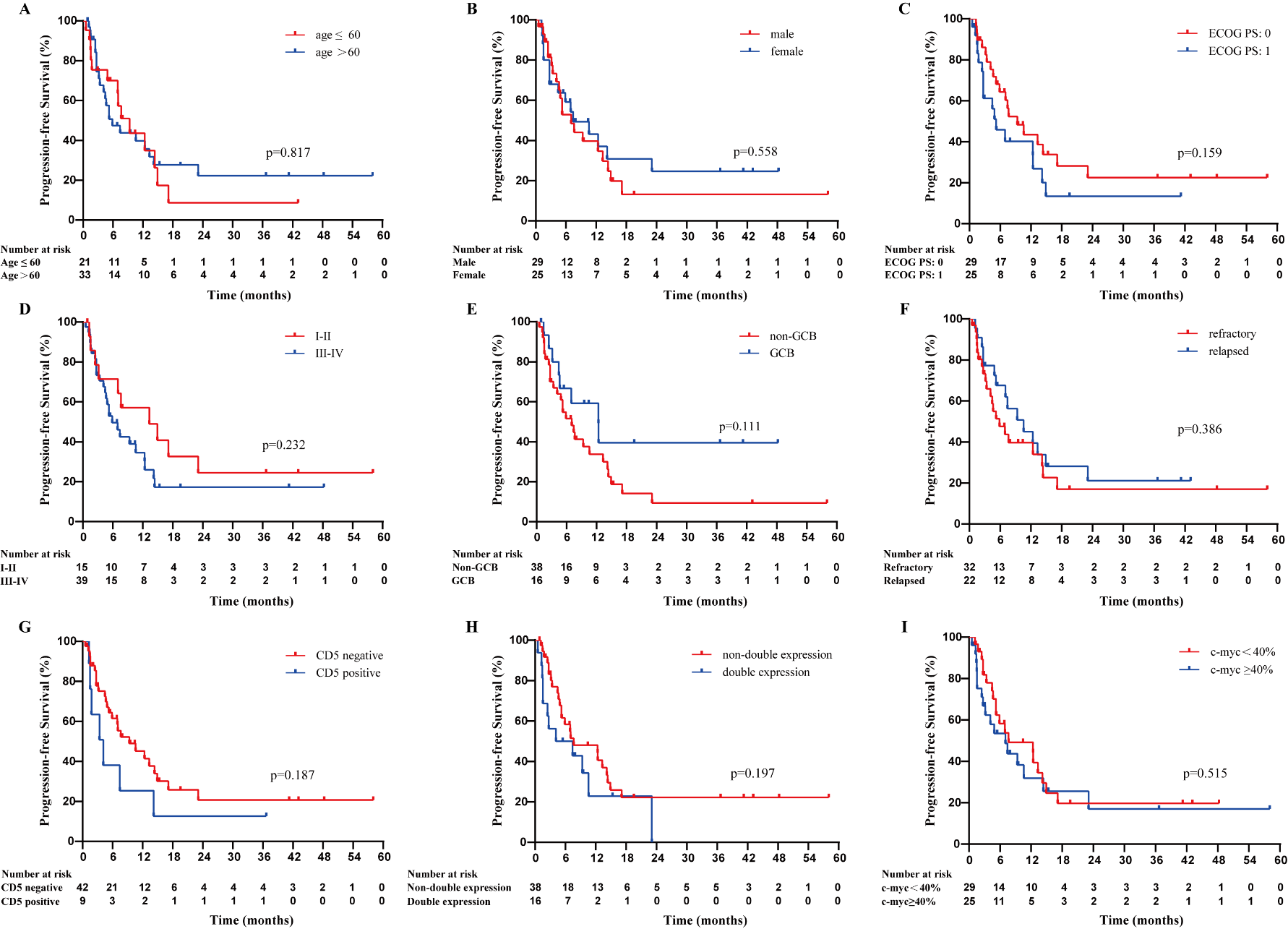


**Figure S3. Subgroup analyses of PFS**.

(A) PFS of patients aged ≤ 60 and >60 years. (B) PFS of patients with male and female. (C) PFS of patients with ECOG PS of 0 and 1. (D) PFS of patients with stage I-II and III-IV. (E) PFS of patients with GCB and non-GCB. (F) PFS of patients with relapsed disease and refractory disease. (G) PFS of patients with CD5 negative and CD5 positive. (H) PFS of patients with non-double expression and double expression. (I) PFS of patients with c-myc level <40% and ≥40%.

Abbreviations: ECOG PS, Eastern Cooperative Oncology Group Performance Status; GCB, germinal center B cell; PFS, progression-free survival.


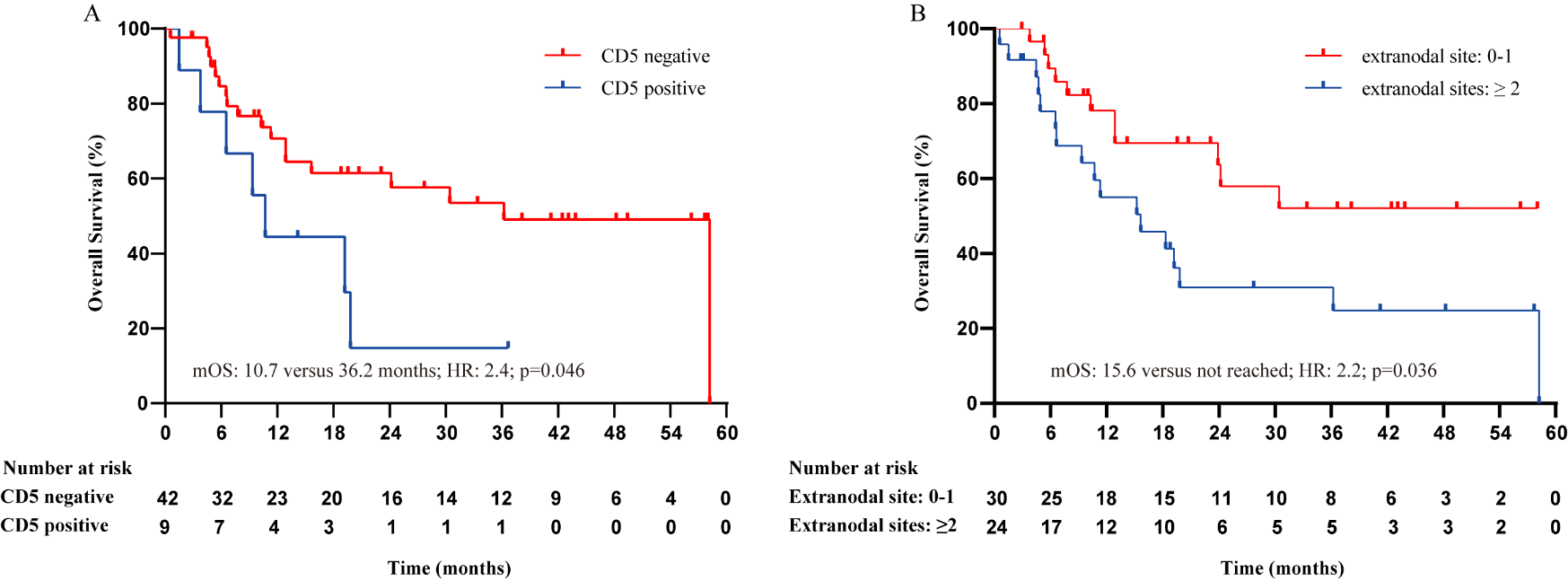


**Figure S4. Subgroup analyses of OS**.

(A) OS of patients with CD5 negative and CD5 positive. (B) OS of the patients with involvement of 0-1 versus ≥ 2 extranodal sites.

Abbreviations: HR, hazard ratio; mOS, median overall survival.


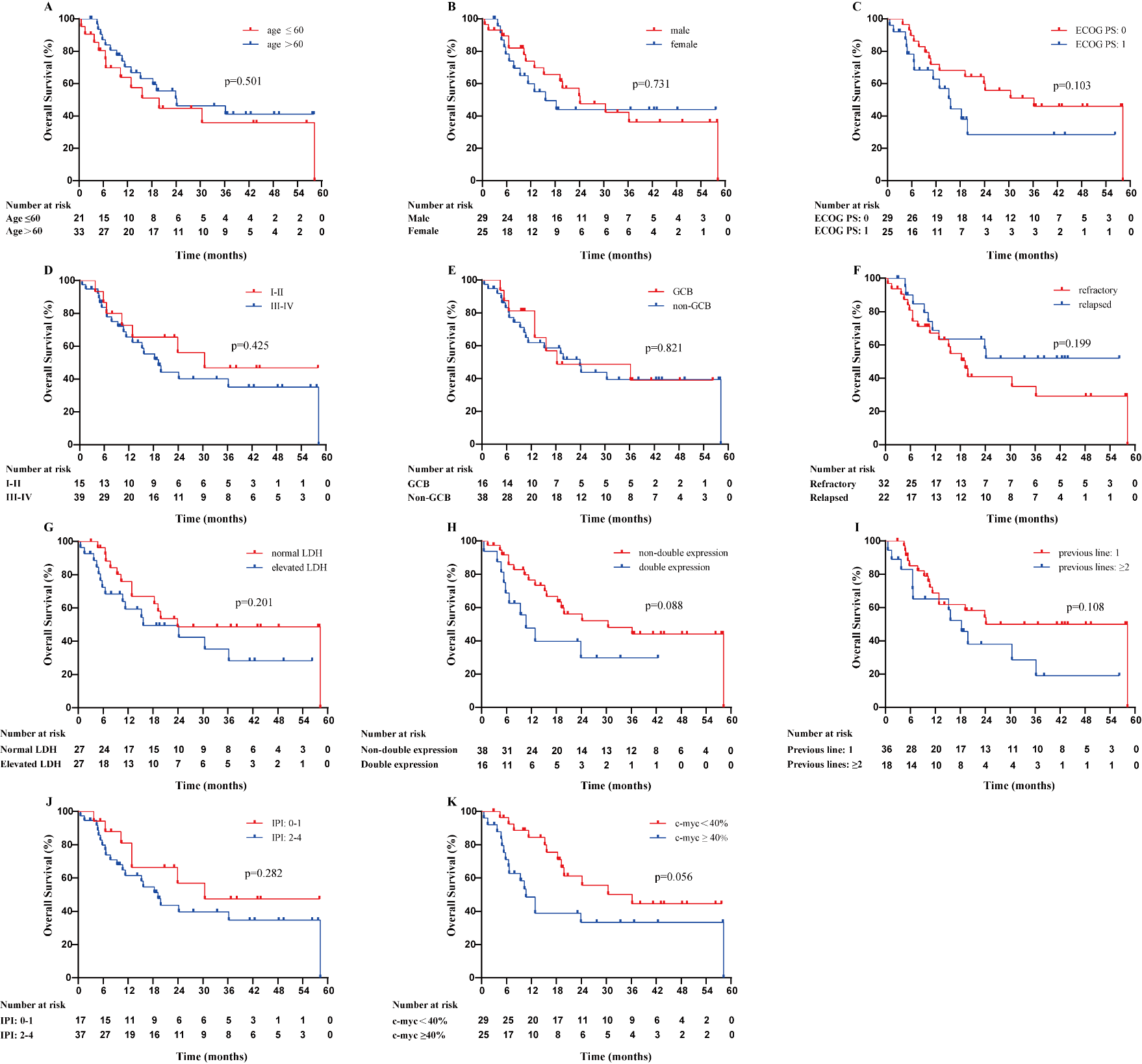


**Figure S5. Subgroup analyses of OS**.

(A) OS of patients aged ≤ 60 and >60 years. (B) OS of patients with male and female. (C) OS of patients with ECOG PS of 0 and 1. (D) OS of patients with stage I-II and III-IV. (E) OS of patients with GCB and non-GCB. (F) OS of patients with relapsed disease and refractory disease. (G) OS of the patients with normal versus elevated LDH. (H) OS of patients with non-double expression and double expression. (I) OS of the patients with 1 versus ≥ 2 previous lines of therapy. (J) OS of the patients with 0-1 versus 2-4 IPI. (K) OS of patients with c-myc level <40% and ≥40%.

Abbreviations: ECOG PS, Eastern Cooperative Oncology Group Performance Status; GCB, germinal center B cell; IPI, International Prognostic Index; LDH, lactate dehydrogenase; OS, overall survival.


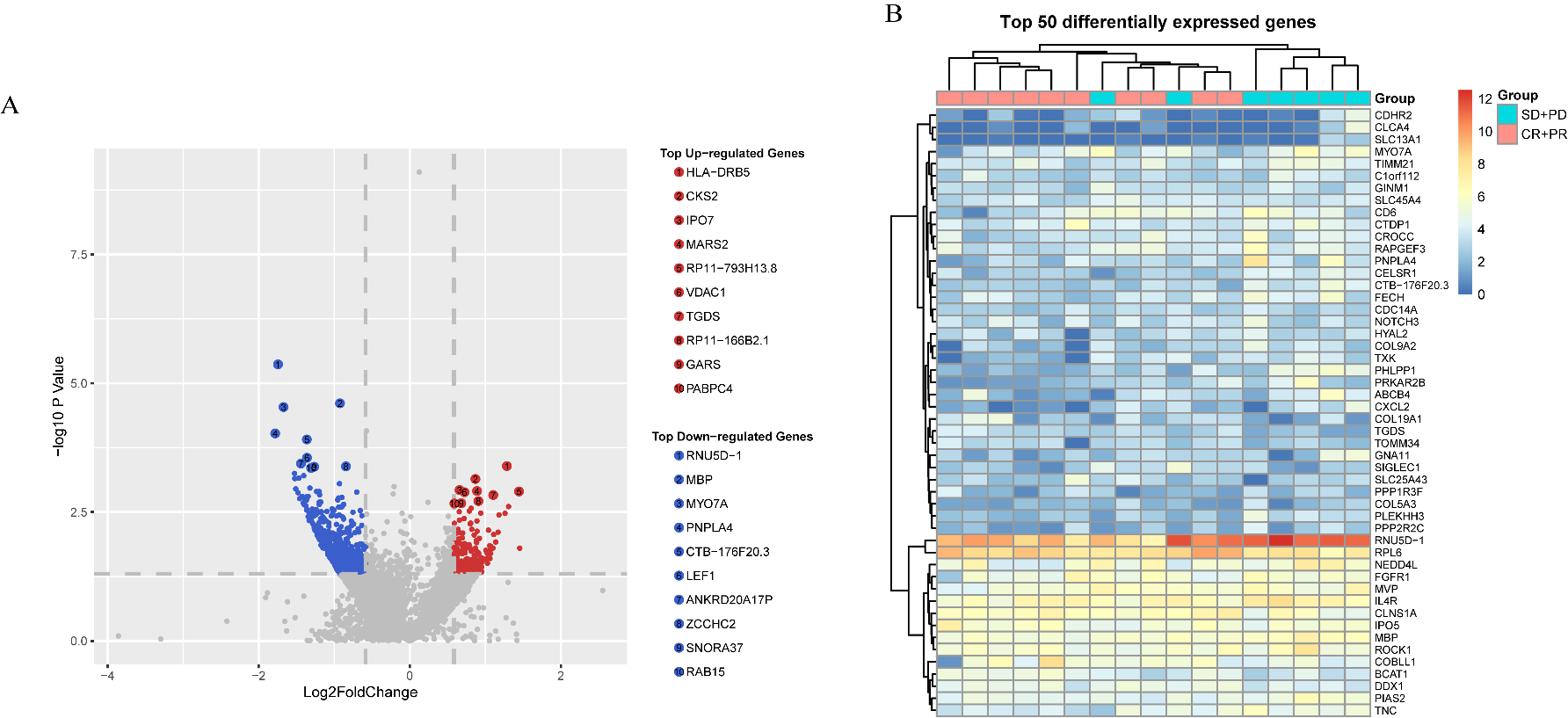


**Figure S6. Transcriptomic analyses.**

(A) Volcano plot of differential gene expression. (B) Heatmap of differential gene expression.

Abbreviations: CR, complete response; PD, progressive disease; PR, partial response; SD, stable disease.
